# Effect of aridity on the β-diversity of alpine soil potential diazotrophs: insights into community assembly and co-occurrence patterns

**DOI:** 10.1128/msystems.01042-23

**Published:** 2023-12-07

**Authors:** Shilong Lei, Xiangtao Wang, Jie Wang, Lu Zhang, Lirong Liao, Guobin Liu, Guoliang Wang, Zilin Song, Chao Zhang

**Affiliations:** 1 The Research Center of Soil and Water Conservation and Ecological Environment, Chinese Academy of Sciences and Ministry of Education, Yangling, Shaanxi, China; 2 Institute of Soil and Water Conservation, Chinese Academy of Sciences and Ministry of Water Resources, Yangling, Shaanxi, China; 3 University of Chinese Academy of Sciences, Beijing, China; 4 College of Animal Science, Tibet Agriculture and Animal Husbandry University, Nyingchi, China; 5 College of Forestry, Guizhou University, Guiyang, China; 6 State Key Laboratory of Soil Erosion and Dryland Farming on the Loess Plateau, Northwest A&F University, Yangling, Shaanxi, China; 7 Institute of Soil and Water Conservation, Chinese Academy of Science, Yangling, Shaanxi, China; 8 College of Natural Resources and Environment, Northwest A&F University, Yangling, Shaanxi, China; CNRS Delegation Alpes, Lyon, Rhône-Alpes, France

**Keywords:** diazotrophs, co-occurrence patterns, alpine ecosystem, climate changes

## Abstract

Microbial diversity plays a vital role in the maintenance of ecosystem functions. However, the current understanding of mechanisms that shape microbial diversity along environmental gradients at broad spatial scales is relatively limited, especially for specific functional groups, such as potential diazotrophs. Here, we conducted an aridity-gradient transect survey from 60 sites across the Tibetan Plateau, the largest alpine ecosystem of the planet, to investigate the ecological processes (e.g., local species pools, community assembly processes, and co-occurrence patterns) that underlie the β-diversity of alpine soil potential diazotrophic communities. We found that aridity strongly and negatively affected the abundance, richness, and β-diversity of soil diazotrophs. Diazotrophs displayed a distance-decay pattern along the aridity gradient, with organisms living in lower aridity habitats having a stronger distance-decay pattern. Arid habitats had lower co-occurrence complexity, including the number of edges and vertices, the average degree, and the number of keystone taxa, as compared with humid habitats. Local species pools explained limited variations in potential diazotrophic β-diversity. In contrast, co-occurrence patterns and stochastic processes (e.g., dispersal limitation and ecological drift) played a significant role in regulating potential diazotrophic β-diversity. The relative importance of stochastic processes and co-occurrence patterns changed with increasing aridity, with stochastic processes weakening whereas that of co-occurrence patterns enhancing. The genera *Geobacter* and *Paenibacillus* were identified as keystone taxa of co-occurrence patterns that are associated with β-diversity. In summary, aridity affects the co-occurrence patterns and community assembly by regulating soil and vegetation characteristics and ultimately shapes the β-diversity of potential diazotrophs. These findings highlight the importance of co-occurrence patterns in structuring microbial diversity and advance the current understanding of mechanisms that drive belowground communities.

**IMPORTANCE:**

Recent studies have shown that community assembly processes and species pools are the main drivers of β-diversity in grassland microbial communities. However, co-occurrence patterns can also drive β-diversity formation by influencing the dispersal and migration of species, the importance of which has not been reported in previous studies. Assessing the impact of co-occurrence patterns on β-diversity is important for understanding the mechanisms of diversity formation. Our study highlights the influence of microbial co-occurrence patterns on β-diversity and combines the drivers of community β-diversity with drought variation, revealing that drought indirectly affects β-diversity by influencing diazotrophic co-occurrence patterns and community assembly.

## INTRODUCTION

Microbial diversity is essential for maintaining ecosystem functions (e.g., climate mediation, aboveground productivity, and soil fertility) (1–3). However, such benefits have been threatened by unprecedented losses in microbial diversity, owing to globally increasing aridity (4–6). Soil diazotrophic communities, which are the most significant source of N in natural ecosystems and play a significant role in the fixation of atmospheric N_2_ (7), are significantly impacted by the aridity level (8). Aridity directly alters plant and soil attributes (plant biomass, soil moisture level, organic substance input, and redox potential), which in turn affects the ecological processes (e.g., species pools, assembly processes, and co-occurrence patterns) of diazotrophic communities and ultimately indirectly influences biogeographic patterns (β-diversity) (9). Clarifying how aridity affects the diversity and structure of potential diazotrophs is crucial for a full comprehension of the causes and impacts of environmental changes on the functioning of terrestrial ecosystems (10–13).

Regional species pools (γ-diversity) have been widely recognized as an underlying determining factor of β-diversity (10). A larger species pool offers a greater number of candidate species available for colonization, which can lead to the establishment of more diverse biotic interactions within the community, leading to higher β-diversity (9, 14). Some studies investigating microbial biogeographic patterns highlighted the roles of assembly processes and argued that microbial community assembly is affected by both deterministic processes (environmental selection) and stochastic processes (dispersal and drift) (14–16). For example, one of the most influential deterministic processes in the assembly of soil bacterial and fungal communities is the environmental filter, which involves a variety of environmental variables like the emergence or survival of species in a specific area (17–19). Stochastic processes, which include ecological drift (random birth and random extinction), dispersal limitation (limitations or obstacles encountered by species during spatial diffusion or migration), and homogenizing dispersal (species diffusion or migration leads to community homogenization) (20), also have a significant impact on how microbial communities are distributed and produce species composition patterns that are similar to randomly generated patterns (21). The co-occurrence patterns uncover how organisms co-occurr in a special environment and influence the colonization of microorganisms and their response to the environment through competition and collaboration, thereby affecting β-diversity (9, 22, 23). Microbial co-occurrence patterns have recently been reported to be potential drivers of microbial β-diversity patterns (10, 24), especially in stressful environments, where the limited niche space would lead to low species richness and fierce competition between microbial taxa, particularly when their requirement of resources is the same (10). However, the relative importance of microbial co-occurrence patterns in the diversity and composition of microbial communities remains poorly understood compared with species pools and community assembly, particularly in fragile ecosystems where biodiversity is vulnerable to environmental changes.

Alpine ecosystems, which occur above the tree line in montane environments, cover ~20% of the earth’s terrestrial surface area and play a vital role in keeping global climate balance and in biodiversity conservation (25–27). These ecosystems are vulnerable and sensitive to climate change owing to extreme environmental stresses (e.g., low temperatures, high winds, and low oxygen) and are one of the most understudied terrestrial ecosystems because of their remoteness and inaccessibility (28). The soil microbial diversity in alpine ecosystems varies significantly owing to the heterogeneity of climate and biogeography, and this variation can be strengthened under climate changes (29). An alpine ecosystem is thus an ideal region to investigate microbial biogeographic patterns along the climate gradient. Because N limitation varied with water limitation in alpine ecosystems (30), clarifying the potential diazotrophic biogeographic patterns along the aridity gradient is essential for the prediction of climate-induced environmental impacts (7).

Here, we conducted a transect soil survey from 60 sites spanning an aridity gradient (−0.2 to 1.0) across the Tibetan Plateau, the largest terrestrial alpine ecosystem of the planet, to investigate the β-diversity patterns of soil potential diazotrophs and to reveal their potential drivers. We hypothesized that (i) assembly processes, especially deterministic processes, are more important in shaping potential diazotrophic β-diversity in more arid environments because water deficiency restricts primary productivity and thus restricts soil resources in arid habitats (30) and that stochastic processes, such as microbial dispersion, are more important in less arid environments; (ii) species pools and co-occurrence patterns also shape the potential diazotrophic β-diversity; and (iii) increasing aridity changes the relative importance of assembly processes, species pools, and co-occurrence patterns to β-diversity.

## RESULTS

### Potential diazotroph community diversity and structure

**Fig 1:**
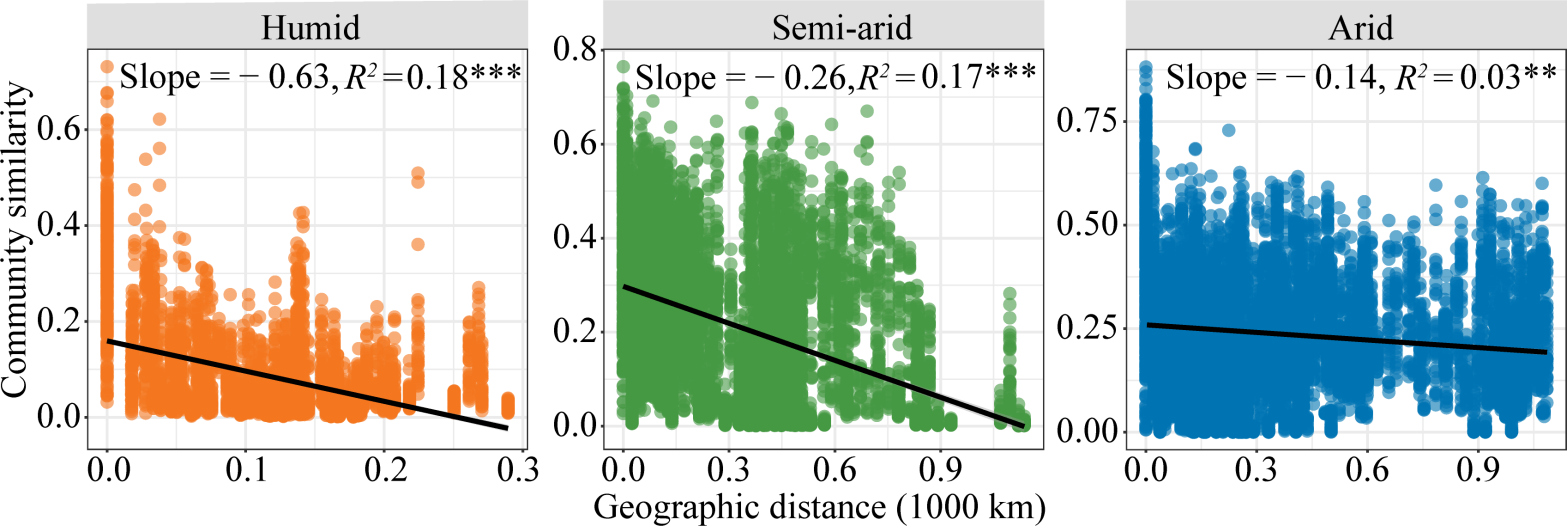
Distant-decay relationships showing the Bray-Curtis similarity of the potential diazotrophic community against geographic distances. Solid lines denote the ordinary least squares linear regressions. *** indicates the significance at *P* < 0.001, and ** indicates the significance at *P* < 0.01.

A total of 6,010,201 diazotroph sequences were assigned to 15,195 operational taxonomic units (OTUs) and then grouped into 28 bins based on phylogenetic relationships (Fig. S2). Alphaproteobacteria accounted for most (mean = 59.0%) of the *nif*H sequences, followed by Deltaproteobacteria (5.4%), Opitutae (2.8%), and Betaproteobacteria (2.4%; Fig. S2). Community similarity was negatively associated with geographic distance and exhibited significant distance-decay relationships (Fig. 1). However, the slopes of the distance-decay curves varied among habitat types (Fig. 1), with steeper slopes observed in humid habitats (−0.63) than in semi-arid or arid habitats (−0.26 and −0.14, respectively; *P* < 0.001; Fig. 1).

**Fig 2:**
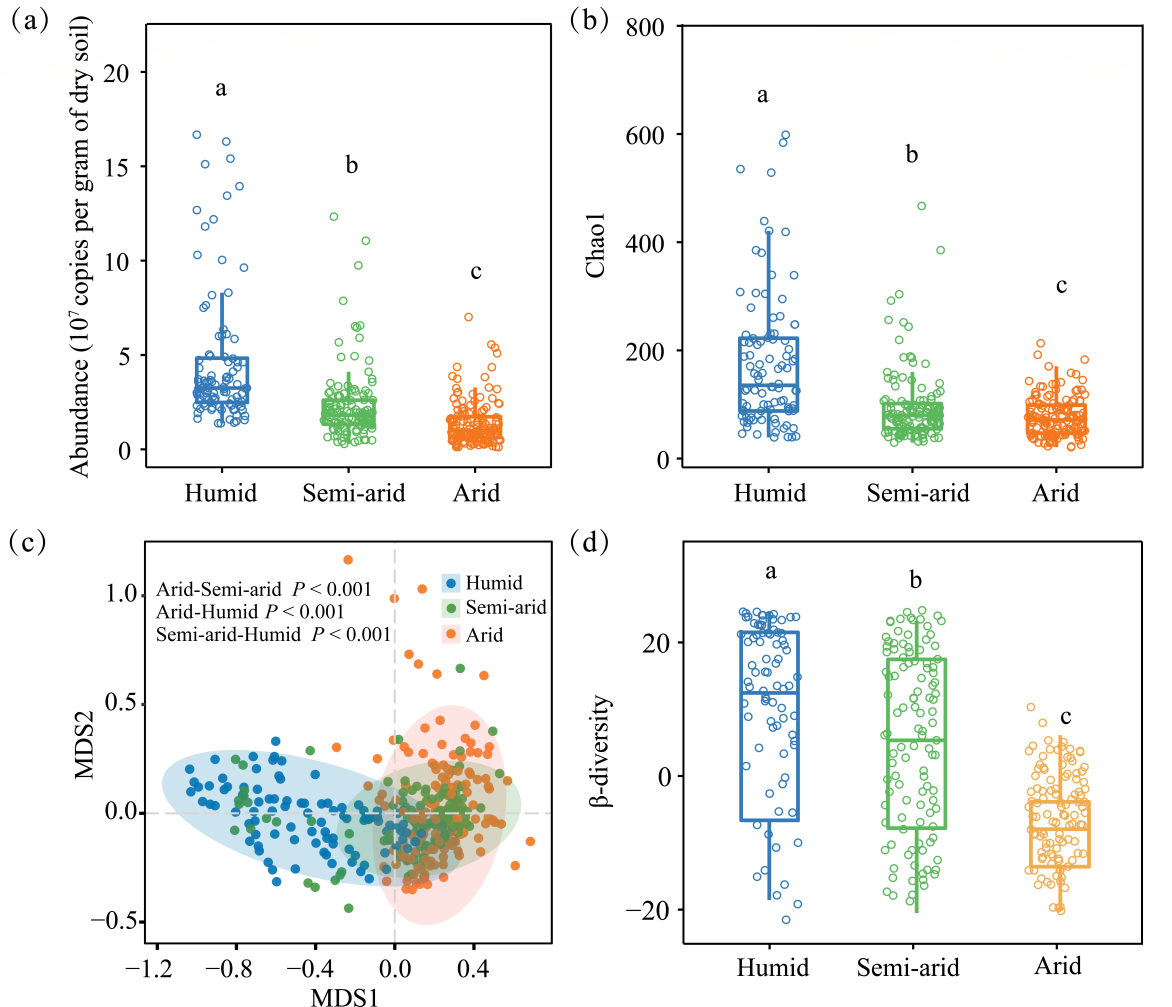
The abundance (**a**) and Chao1 estimator (**b**) of the potential diazotrophic community at different aridity habitats. Community dissimilarity in the potential diazotrophic community (**c**) based on the nonmetric multidimensional scaling (NMDS). Shaded areas indicate 95% confidence intervals of fit. The dissimilarity variation (**d**) between different aridity gradients based on Bray-Curtis dissimilarity. In the box plots, the upper boundary of each box indicates the 25th percentile, the horizontal line inside each box marks the median, and the lower boundary of the box indicates the 75th percentile. Different letters indicate significant differences (*P* < 0.05) by Tukey’s test.

Both diazotrophic richness (i.e., Chao1 estimator) and abundance (i.e., quantitative PCR [qPCR]-based copy number) were decreased with site aridity (Fig. 2a and b), and the results of the nonmetric multidimensional scaling analysis indicated that diazotrophic community structure shifted along the aridity gradient (permutational multivariate analysis of variance tests, *P* < 0.01; Fig. 2c; Table 1). In addition, diazotrophic β-diversity was significantly (*P* < 0.05) higher at the humid sites than at either the semi-arid or arid sites (Fig. 2d). The ordinary least squares linear regression model (OLSLRM) results indicated that aridity was significantly and negatively correlated with both α-diversity and β-diversity, not γ-diversity, of the diazotrophic communities (Fig. S3).

**TABLE 1:** Results of permutational multivariate analysis of variance tests for potential diazotrophic communities among different aridity habitats^*a*^

Community	Bray-Curtis	
	*F*	*P*
Arid–semi-arid	2.913	0.011^**^
Arid-humid	23.769	0.093^**^
Semi-arid–humid	18.007	0.076^**^

^*a*^ **, *P* < 0.01.

### Local species pool and community assembly

**Fig 3:**
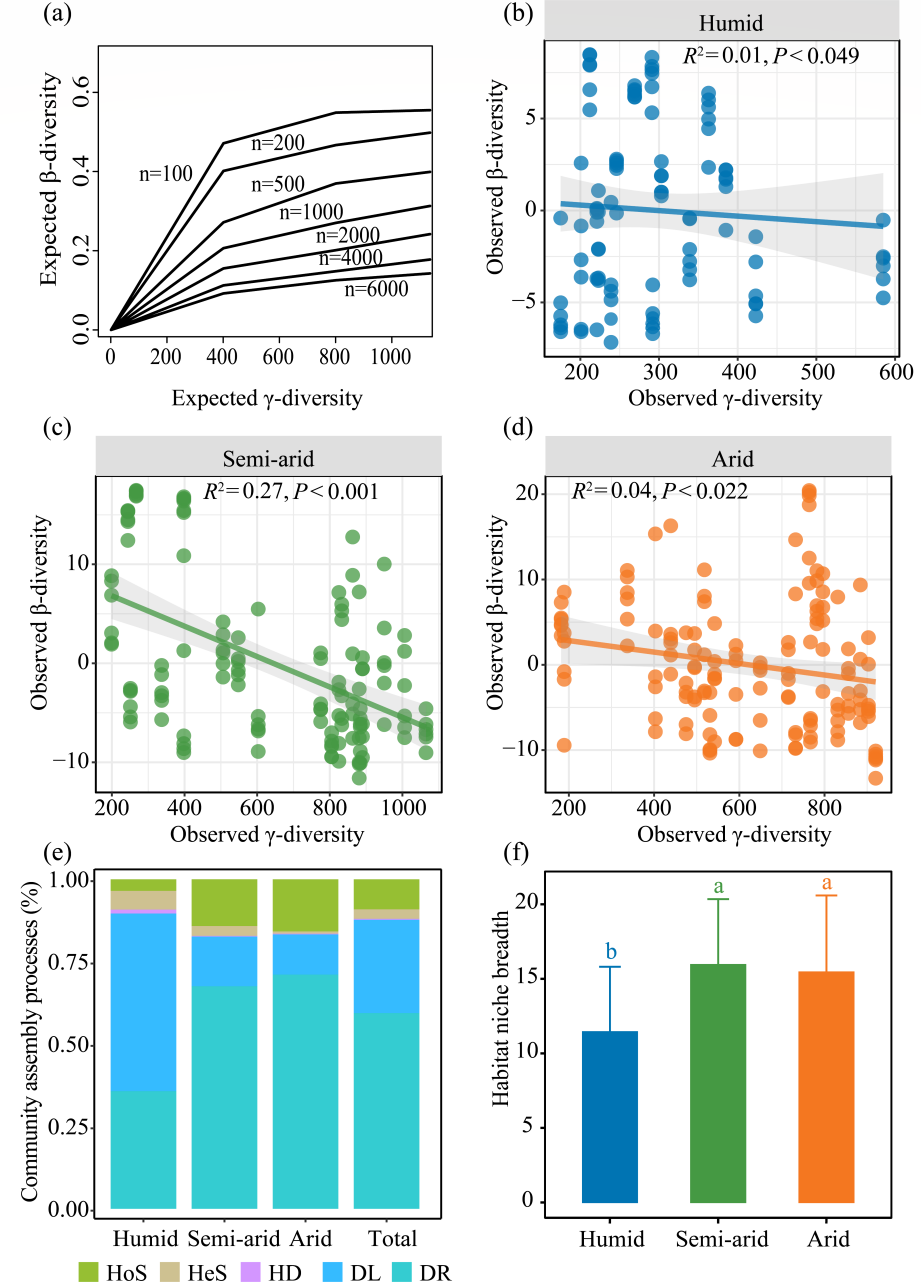
(**a**) Expected relationships between β-diversity and species pool (γ-diversity) when *n* individuals are drawn from a species pool of a fixed size. The “*n*” in panel a is the number of individuals in each plot. Observed relationships between β-diversity and species pool (γ-diversity) in humid (**b**), semi-arid (**c**), and arid (**d**) habitats. Gray shaded areas indicate 95% confidence intervals of fit. (**e**) Relative contributions of the ecological processes that governed potential diazotrophic community assembly based on the iCAMP model and (**f**) the habitat niche breadths in three aridity habitats. Different letters indicate significant differences (*P* < 0.05) by Tukey’s test. HoS, homogeneous selection; HeS, heterogeneous selection; HD, homogenizing dispersal; DL, dispersal limitation; DR, drift.

**Fig 4:**
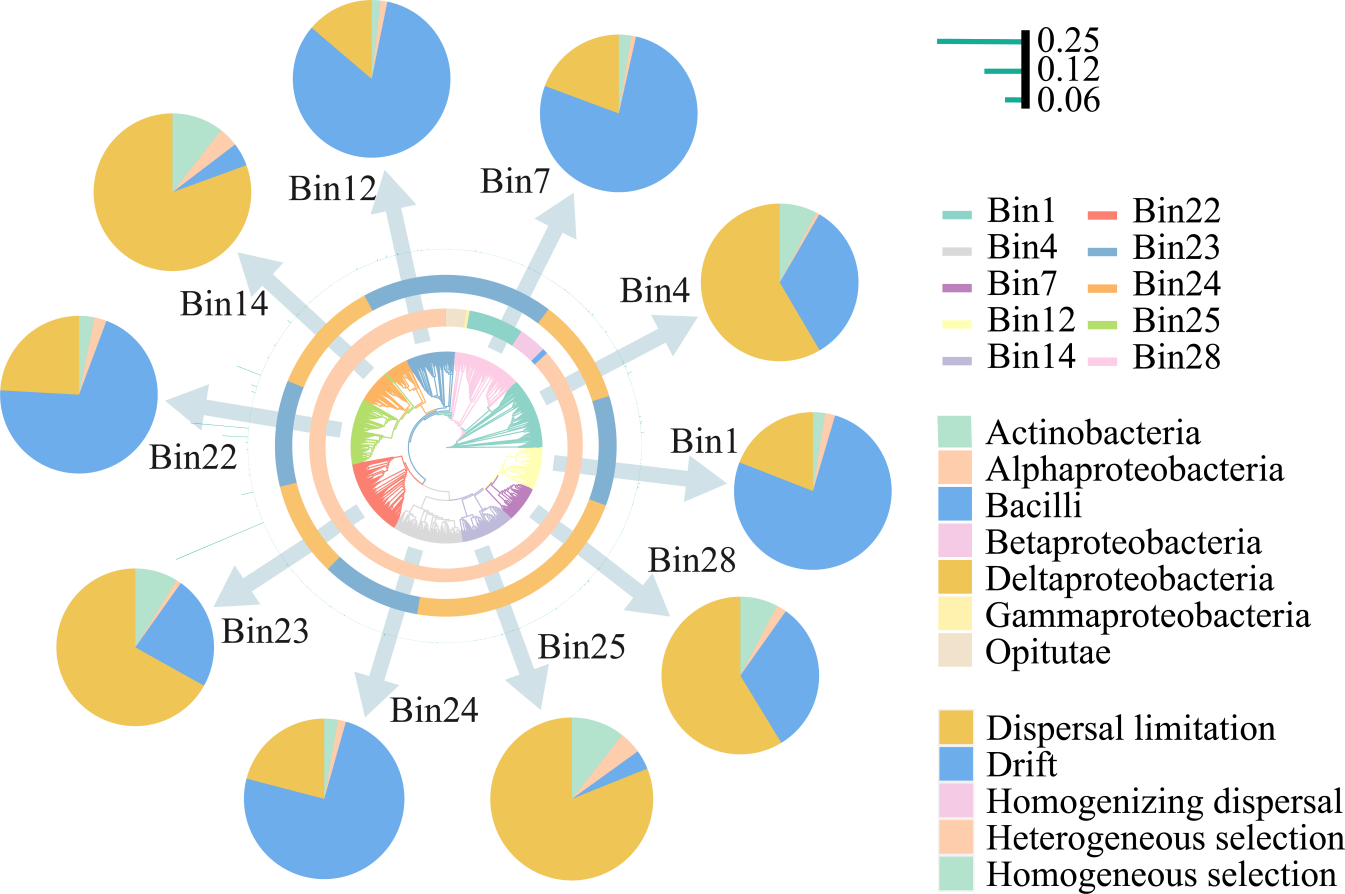
Variations in ecological processes across dominant potential diazotrophic taxa based on the iCAMP model. The center is the phylogenetic tree of the 10 bins. The class level of each bin is indicated in the first annulus; major ecological processes governing dominant nitrogen-fixing microorganism groups are in the second annulus; the relative abundance of each OTU is in the third annulus; and pie charts show the contribution of each bin to the different ecological processes.

The simulation model revealed that the expected β-diversity increased with increasing γ-diversity, regardless of the number of individuals (Fig. 3a). However, contrary to our hypothesis (ii), which suggested that β-diversity is affected by the species pool, the relationship between the observed γ-diversity and the observed β-diversity did not follow the expected pattern (Fig. 3b through d), which indicated that the species pool has little influence on the potential diazotrophic β-diversity in alpine ecosystems. The results of the phylogenetic bin-based null model analysis (iCAMP) suggested that stochastic processes, mainly dispersal limitation and ecological drift, made the largest donation to diazotrophic community assembly, together accounting for 87.7% of the total variance in diazotrophic community composition, whereas heterogeneous and homogenous selection accounted for only 2.9% and 9.1%, respectively (Fig. 3e). Furthermore, the role of dispersal limitation decreased with increasing aridity, whereas that of ecological drift increased (Fig. 3e). The neutral community model indicated that the species migration rate in arid habitats was relatively low (Table 2) but had a greater environmental niche breadth (Fig. 3f). A total of 15,195 OTUs were classified into 28 bins based on phylogenetic relationships. Ten OTU bins that were most strongly affected by assembly processes were selected, including five bins regulated by dispersal limitation (group 1) and five regulated by ecological drift (group 2, Fig. 4). The two groups contributed 61% and 74% to the drift and dispersal limitation (Fig. 4). The most abundant taxa contained Actinobacteria, Alphaproteobacteria, Betaproteobacteria, Deltaproteobacteria, and Opitutae (Fig. 4).

**TABLE 2:** Fit of the neutral model in potential diazotrophic communities in three aridity gradients^*a*^^,^^*b*^

Humid		Semi-dry		Dry	
*m*	*R* ^2^	*m*	*R* ^2^	*m*	*R* ^2^
0.007	0.292	0.0022	0.509	0.0008	0.541

^*a*^ Note. *m*, migration rates.

^*b*^ *R*^2^ and *m* values indicate the fit to the neutral model and the estimated migration rate, respectively.

### Co-occurrence network patterns

**Fig 5:**
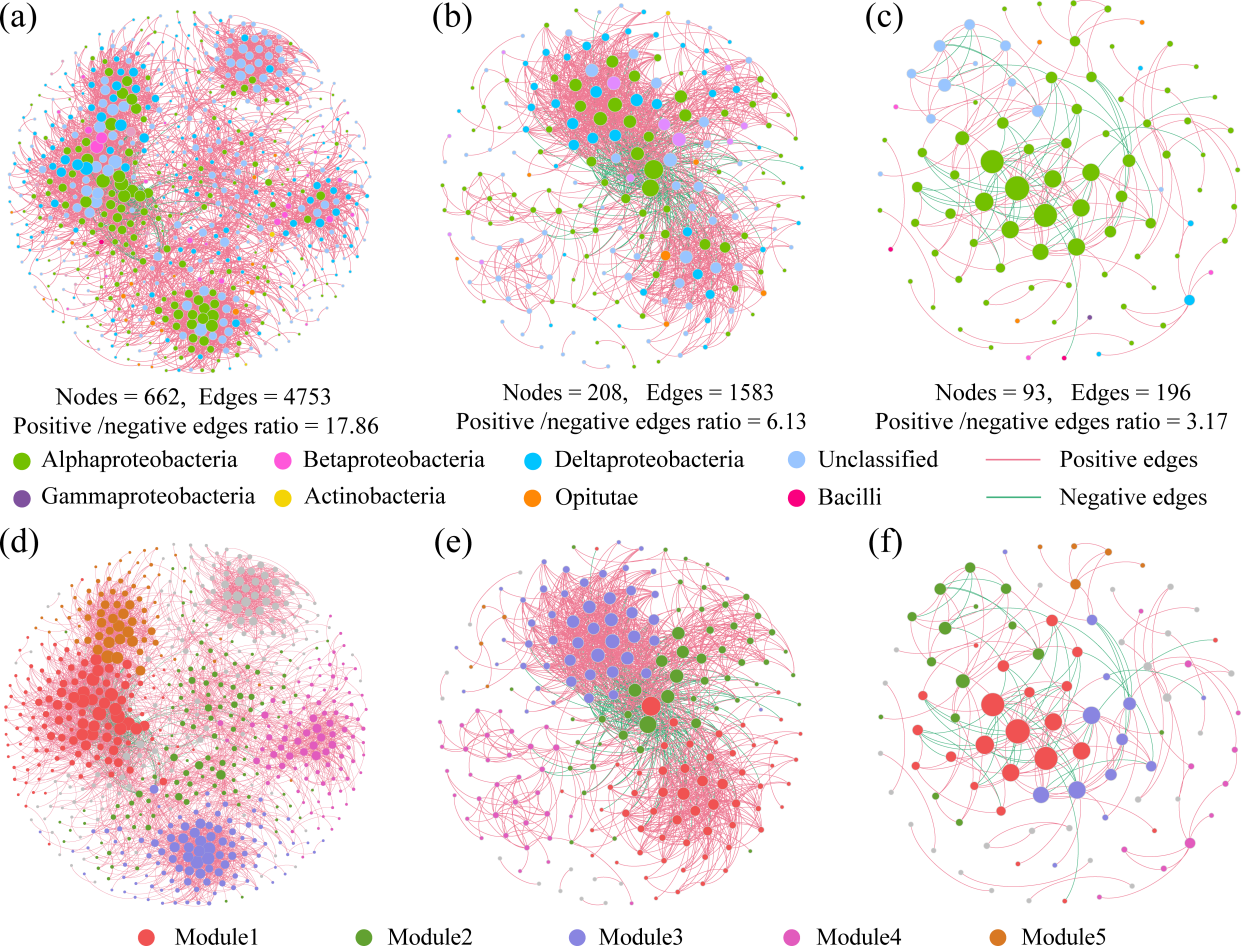
The potential diazotrophic co-occurrence networks are colored by potential diazotrophic classes (**a to c**) and by modules (**d to f**). The networks in panels a and d were in humid habitats, those in panels b and e were in semi-arid habitats, and those in panels c and f were in arid habitats. The size of each vertex is proportional to the number of connections (degree), and the thickness of each connection between two vertices (edges) is proportional to the value of Spearman’s correlation coefficients. Modules 1–5 in humid, semi-arid, and arid habitats were the five clusters of closely interconnected vertices. The green edges in all figures indicate negative interactions between two potential diazotrophic vertices, while red edges indicate positive interactions.

The co-occurrence networks across the three habitats followed a power-law distribution pattern (Fig. S4). The humid, semi-arid, and arid habitat networks captured 4,753 edges among 662 vertices, 1,583 edges among 208 vertices, and 196 edge counts among 93 vertices (Fig. 5a through c). Among the subnetworks, the number of edges (NE), number of vertices (NV), and average degree (AD) significantly decreased with increasing aridity (*P* < 0.05; Fig. S5a through c), and the density (DEN), clustering coefficient (CC), average path length (APL), and modularity (MOD) in semi-arid habitats were significantly lower than those in humid and arid habitats (*P* < 0.05; Fig. S5d through g). In addition, keystone taxa (potential diazotroph module and connector hubs) in the vertices were affiliated with Alphaproteobacteria (47 OTUs), Deltaproteobacteria (48 OTUs), Betaproteobacteria (9 OTUs), and Opitutae (6 OTUs) in humid habitats; Alphaproteobacteria (24 OTUs), Deltaproteobacteria (6 OTUs), Betaproteobacteria (3 OTUs), and Opitutae (2 OTUs) in semi-arid habitats; and Alphaproteobacteria (25 OTUs), Deltaproteobacteria (3 OTUs), Betaproteobacteria (2 OTUs), Bacilli (2 OTUs), and Opitutae (2 OTUs) in arid habitats (Fig. S6a through c). The arid co-occurrence networks harbored the lowest positive/negative edge ratios among the three habitats (Fig. 5a through c). Since the positive and negative edges of co-occurrence networks may be indicative of species cooperation and competition, the lower positive/negative edge ratio probably indicated stronger species competition in arid habitats.

**Fig 6:**
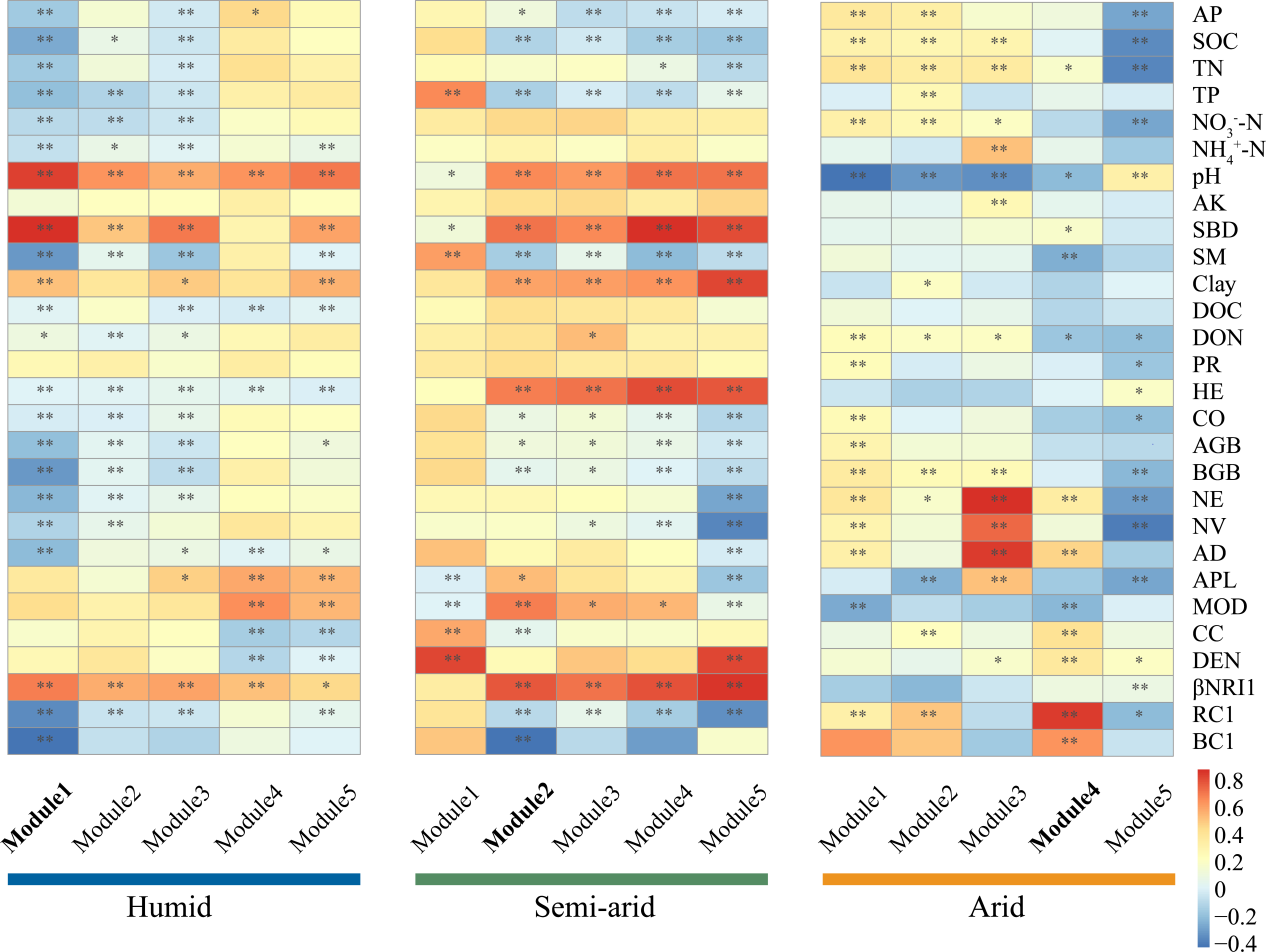
Spearman’s correlation between soil properties, plant characteristics, topological features, and assembly processes and modules of co-occurrence networks in different aridity habitats. The color of the square represents positive (red) and negative (blue). AL, altitude; LO, longitude; LA, latitude; ARI, aridity; MAT, mean annual temperature; SBD, soil bulk density; Clay, soil texture; SOC, soil organic content; SM, soil moisture; TP, total phosphorus; AP, available phosphorus; TN, total nitrogen; NO_3_^−^-N, nitrate nitrogen; NH_4_^+^-N, ammonium nitrogen; BGB, belowground biomass; AGB, aboveground biomass; CO, coverage; HE, height; βNRI1, the first axis of the principal component analysis (PCA) of the within-bin beta Net Relatedness Index; RC1, the first axis of the PCA of the modified Raup-Crick metric; BC1, the first axis of the PCA of the Bray-Curtis matrix; NE, the number of edges; NV, the number of vertices; AD, average degree; APL, average path length; MOD, modularity; CC, clustering coefficient; DEN, density.

The OLSLRM between network topological features and β-diversity showed that the subnetwork topological features of potential diazotrophs were significantly related to their β-diversity (*P* < 0.05; Fig. S7). Topological features, such as NV, NE, APL, and MOD, were significantly and positively related to β-diversity (*P* < 0.05; Fig. S7). These findings demonstrated that a longer path length and loose connectivity in the network would lead to a heterogeneous diazotrophic community, which is consistent with our hypothesis (ii) that indicated that co-occurrence patterns contribute to diversity. Co-occurrence networks were further divided into smaller coherent modules, and their eigengenes were strongly correlated with pH (Fig. 5d through f and 6). Diazotrophic β-diversity was negatively correlated with module 1 in the humid habitats and module 2 in the semi-arid habitats and was positively correlated with module 4 in the arid habitats (Fig. 6). The genera *Geobacter* (Deltaproteobacteria) and *Paenibacillus* (Bacilli) were identified as keystone taxa (Table 3), which indicated a significant association between the connected module members and diazotrophic β-diversity (Table 3).

**TABLE 3:** The keystone taxa significantly associated with module species diversity and community β-diversity in the potential diazotrophic networks in different aridity habitats^*a*^

Network	OTU ID	Module	Class/genus	Diversity	β-Diversity
Humid	OTU9374	1	Alphaproteobacteria	0.73^**^	0.72^**^
	OTU12322	1	Deltaproteobacteria/*Geobacter*	0.66^**^	0.67^**^
Semi-arid	OTU11202	2	Deltaproteobacteria/*Geobacter*	0.40^**^	0.41^**^
Arid	OTU14365	4	Bacilli/*Paenibacillus*	0.57^*^	0.25^*^

^*a*^ **, *P* < 0.01; *, *P* < 0.05.

### Underlying drivers of potential diazotrophic β-diversity

**Fig 7:**
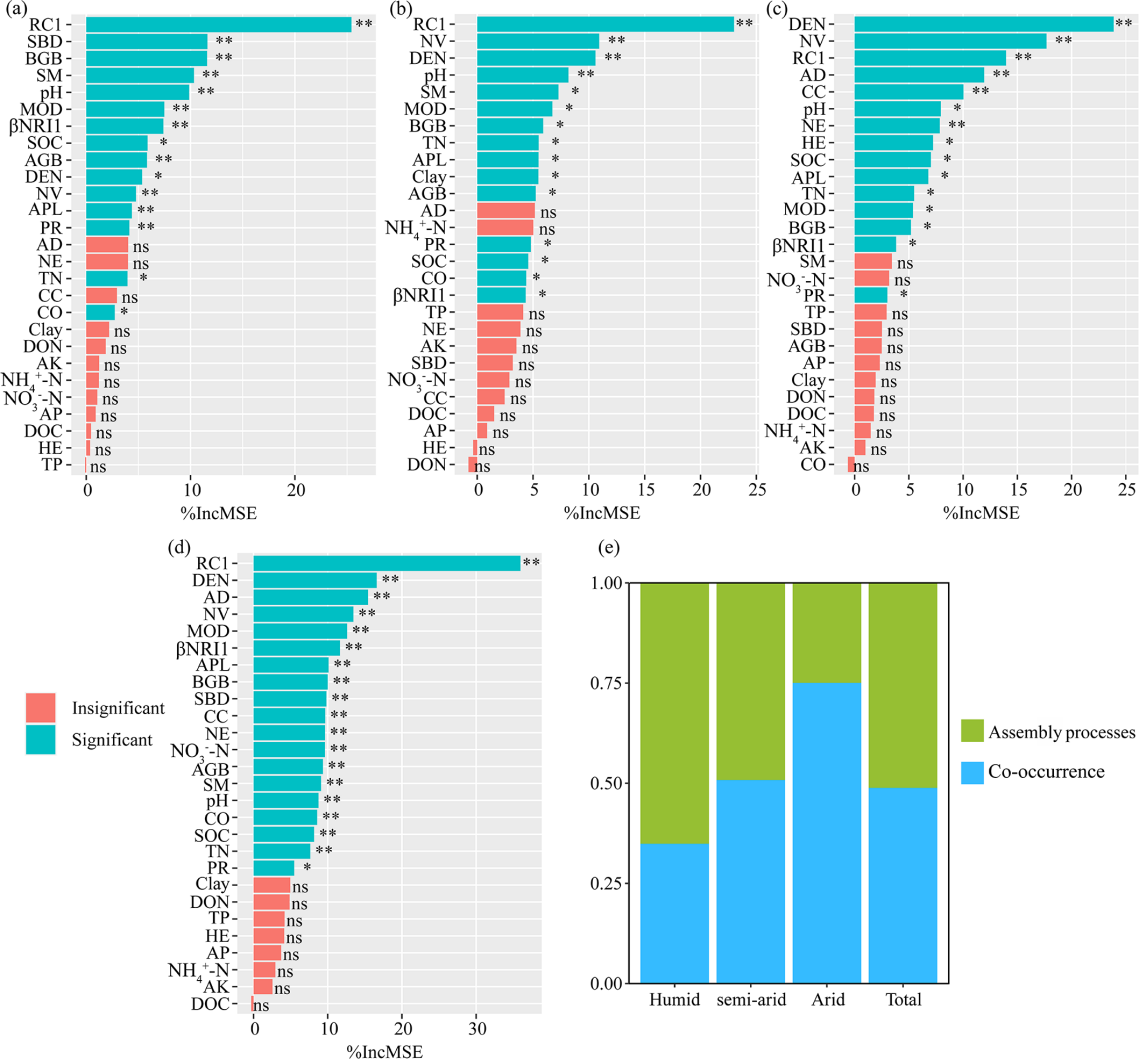
The random forest analysis showed the relative contribution of aridity, soil properties, plant characteristics, assembly processes, and co-occurrence patterns to potential diazotrophic β-diversity in different habitats (**a to c**) and total areas (**d**) (*P < 0.05; **P < 0.01; ns, insignificant). (e) The relative importance of assembly processes and co-occurrence patterns, which were calculated by the increase in mean squared error (%incMSE) of assembly processes (MOD, DEN, and NV in humid habitats; NV, DEN, and MOD in semi-arid habitats; DEN, NV, and AD in arid habitats) and co-occurrence patterns (βNRI1 and RC1) divided by their sum, respectively. SBD, soil bulk density; SOC, soil organic content; Clay, clay content; SM, soil moisture; TP, total phosphorus; AP, available phosphorus; TN, total nitrogen; NO_3_^−^-N, nitrate nitrogen; NH_4_^+^-N, ammonium nitrogen; BGB, belowground biomass; AGB, aboveground biomass; CO, coverage; HE, height; βNRI1, the first axis of the PCA of the within-bin beta Net Relatedness Index; RC1, the first axis of the PCA of the modified Raup-Crick metric; NV, the number of vertices; AD, average degree; APL, average path length; MOD, modularity; DEN, density.

**Fig 8:**
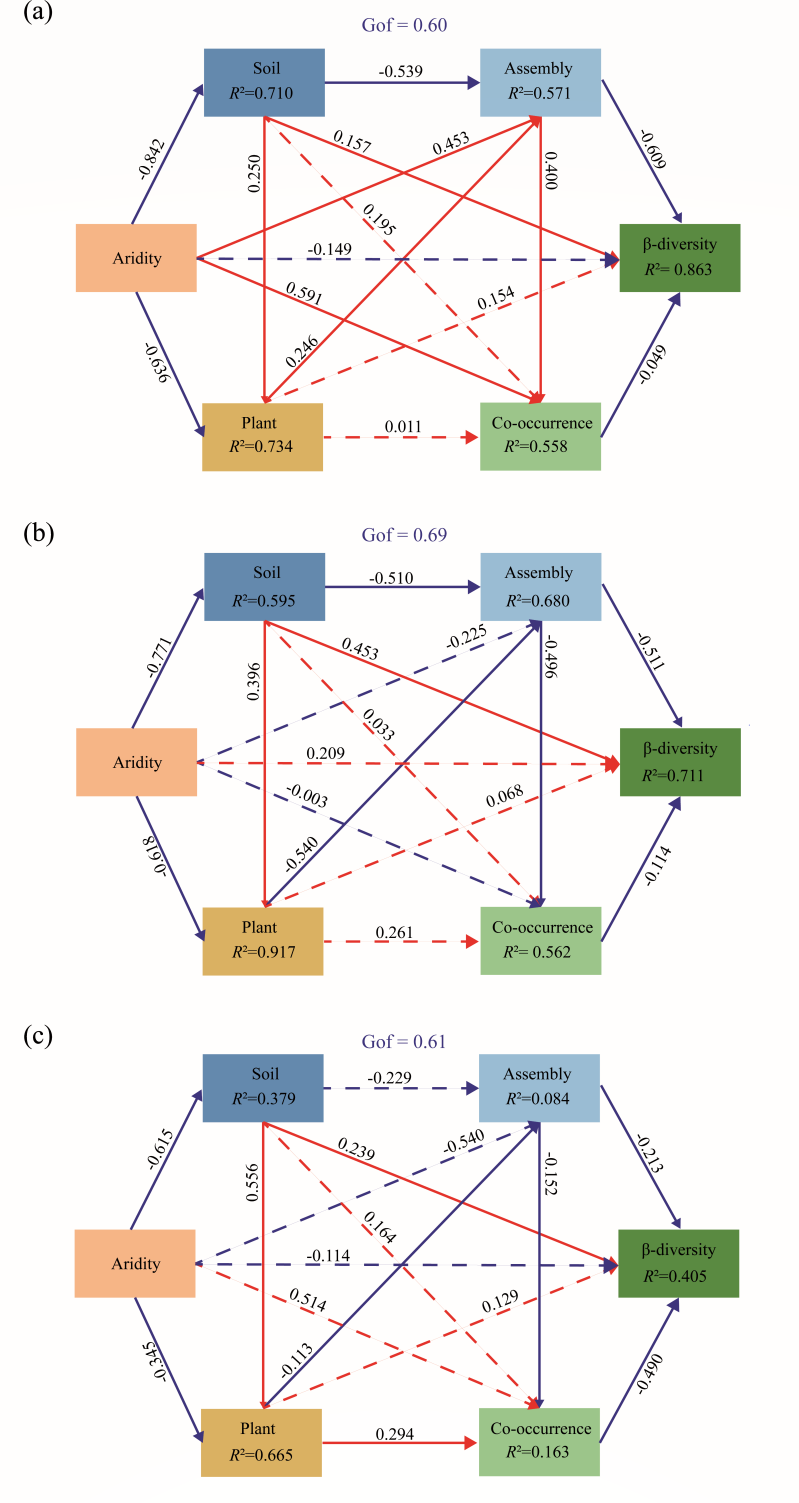
The partial least squares path model (PLS-PM) shows the effects of increasing aridity on potential diazotrophic β-diversity in humid (a), semi-humid (b) and arid (c) habitats. Solid arrows indicate significant relationships, and dashed arrows indicate no significant relationship. Red arrows indicate positive relationships, and blue arrows indicate negative relationships. Numbers are the affecting coefficients. *R*^2^ is the explained variance by the variables. Gof is the proportion of variance explained by the model. The soil properties included soil total nitrogen, soil organic carbon, pH, and soil moisture. The vegetation characteristics included coverage, plant richness, aboveground biomass, and belowground biomass. The assembly processes included the first axis of the PCA of the within-bin beta net relatedness index and the first axis of the PCA of the modified Raup-Crick metric. The co-occurrence patterns included the number of vertices, average path length, modularity, and density. β-Diversity was calculated as the first axis of the PCA of the Bray-Curtis matrix.

The results of the random forest analysis indicated that community assembly (e.g., βNRI1 and RC1) and network topological features (e.g., NE, NV, AD, MOD, APL, CC, and DEN) played greater roles than other predictors in determining diazotrophic β-diversity and, together, accounted for 48.2% of the variance in diazotrophic β-diversity (Fig. 7d). Furthermore, the relative importance of assembly processes decreased with increasing aridity, whereas the importance of network topological features (MOD, DEN, and NV in humid habitats; NV, DEN, and MOD in semi-arid habitats; DEN, NV, and AD in arid habitats) increased (Fig. 7a through c and e). Furthermore, the established partial least squares path model (PLS-PM) explained 86.3%, 71.1%, and 40.5% of the variation in β-diversity in humid, semi-arid, and arid habitats, respectively (Fig. 8). Integrating the contributions of diazotrophic community assembly (path coefficients = −0.609, –0.511, and −0.213 in humid, semi-arid, and arid habitats, respectively; *P* < 0.01; Fig. 8) and co-occurrence patterns (path coefficients = −0.049,–0.114, and −0.490 in humid, semi-arid, and arid environments, respectively; *P* < 0.01; Fig. 8) significantly influenced β-diversity. Soil properties (path coefficients = 0.157, 0.453, and 0.239 in humid, semi-arid, and arid habitats, respectively; *P* < 0.01; Fig. 8) also directly and significantly affected β-diversity, whereas vegetation characteristics and soil properties indirectly and significantly influenced β-diversity by affecting community assembly and co-occurrence networks (*P* < 0.01; Fig. 8). In the three habitats, aridity has no direct and significant impact on β-diversity (Fig. 8). These findings indicate that aridity indirectly shapes β-diversity by influencing soil properties (total nitrogen, soil organic carbon, soil moisture, and pH), vegetation characteristics (coverage, plant richness, aboveground biomass, and belowground biomass), stochastic processes (βNRI1 and RC1), and species co-occurrence patterns (NV, APL, DEN, and MOD). Notably, the path coefficients of the assembly processes decreased (from −0.609 to −0.213) with increasing aridity, whereas those of the co-occurrence network increased (from −0.049 to −0.490; Fig. 8). This finding suggested that the importance of ecological processes in shaping the potential diazotrophic β-diversity appeared to be driven by aridity level, which indicated that aridity changes soil properties, community assemblage processes, and network stability, which is the same as hypothesis (iii).

## DISCUSSION

To the best of our knowledge, this is the first study to report the spatial distribution patterns of potential diazotrophic communities in alpine ecosystems along a large environmental gradient. The findings of the study indicate that global increasing aridity significantly affects the biogeographic patterns of alpine soil potential diazotrophic communities. Distinct potential diazotrophic community compositions and distance-decay relationships were observed at different aridity levels, and increasing aridity appeared to both reduce diazotrophic richness through negative effects on resource inputs (i.e., available C) and alter the β-diversity of diazotrophic communities by regulating the assembly processes and co-occurrence patterns.

### Aridity alters geographical patterns and assembly processes

The distance-decay relationship slopes of the potential diazotrophic communities were remarkably higher (−0.63 to −0.14, Fig. 1) than those reported for the potential diazotrophic communities of southern China (−0.067) (19). This is likely due to the environmental conditions of the Qinghai-Tibet Plateau’s soil, which is typically frozen for more than 180 days per year, with permafrost and seasonally frozen soil jointly accounting for 80% of the total area (31). Indeed, long-term and extensive freezing can limit species migration in soil and can weaken species turnover (7). The potential diazotrophic communities investigated by the present study exhibited steeper distance-decay relationship slopes in humid habitats (fitness *R*^2^ < 0.1) than in semi-arid and arid habitats (Fig. 1), which suggests that the spatial structure and turnover of soil potential diazotrophic communities decreased with increasing aridity. These results support previous reports and can be caused by the physical barrier that aridity (i.e., water stress) imposes on the dispersal capacity of potential diazotrophs in high-aridity habitats (14, 32). Previous studies have reported that soil biota use dormancy as a metabolic strategy to deal with environmental stresses (33), and many microbes (e.g., Firmicutes, Actinobacteria, and Actinobacteriota) can transition from active to dormant states under water-deficient conditions in order to sustain their growth and, thereby, limit species turnover (29).

Stochastic processes indicate an equal chance of dispersal for each species and are mainly dominated by homogenizing dispersal, dispersal limitation, and ecological drift (34). Contrary to our first hypothesis, deterministic processes are more important in shaping diazotrophic β-diversity, and the application of the iCAMP model revealed that stochastic processes (e.g., dispersal limitation and ecological drift), rather than deterministic processes (e.g., heterogeneous and homogeneous selection), play a dominant role in shaping the potential diazotrophic community assembly of alpine soils (Fig. 3e). Diazotrophs, which have smaller bodies, are usually considered to be less influenced by dispersal limitation than larger organisms and have stronger dispersal ability, which is inconsistent with our results (29). The reason could be that the soil freezing of the Tibetan Plateau hindered the migration of microorganisms and increased the physical barrier by reducing the fluidity of water and the activity of microorganisms (35). This weak environmental effect (deterministic processes) may be caused by the dormancy strategies of belowground microorganisms in alpine ecosystems, which could enhance microbial resistance and alleviate selection pressures (28, 29, 36). However, it is important to note that our study also revealed a higher environmental effect in more arid habitats. This finding suggests that aridity can actually enhance the selection effect on the potential diazotrophic community, despite the presence of dormancy strategies that enhance the adaptability of potential diazotrophs.

Although dispersal limitation and ecological drift contribute significantly to diazotrophic β-diversity, the contribution of the two processes shifts along the aridity gradient, with the importance of dispersal limitation decreasing with increasing aridity and the importance of ecological drift increasing (Fig. 3e). This finding may be explained as follows. Microbial dispersal is typically a passive process that involves transportation by air, flow, and hitchhiking (29). However, this passive dispersal process was restricted in the more arid regions of the Tibetan Plateau, not only because of soil freezing, which presents a physical obstacle, but also because the lower water availability hinders microbial movement in the soil, especially for those inactive microorganisms (27, 37). Furthermore, dormancy may increase the resistance of diazotrophs to water stress, resulting in weak long-distance dispersal (29). For instance, Betaproteobacteria from the genus *Polaromonas* were widely dispersed in alpine habitats, and their dormancy might prevent them from spreading from soil to air (38). Previous studies have demonstrated that ecological drift is more important in shaping microbial community structure under weak environmental selection, low microbial richness, and low member abundance, such as in host-associated environments (39, 40). In arid habitats, diazotrophs with low richness and abundance are more vulnerable and sensitive to drift because a slight reduction in their abundance can result in their extinction (41). A larger number of vegetation patches observed in semi-arid and arid habitats (data were not shown) also reinforce the roles of ecological drift in the establishment of the diazotrophic community (42). That is because the lower number of individuals in plant patches should increase the risk of local species extinction and enhance the impact of ecological drift on communities (42, 43). When a species’ population size decreases, it becomes more vulnerable to environmental fluctuations and chance events, as there are fewer individuals available to contribute to the gene pool or ecological interactions (44). Functional redundancy, which refers to the similar or identical functions of different species, appears to be quite high in the microbial communities of stressed environments because redundant species are needed to maintain ecological functionality in the face of species extinction (45). Consequently, in arid areas with strong functional redundancy, microorganisms become more susceptible to ecological drift. This susceptibility arises from the reduced selective pressures and increased stochasticity associated with functional redundancy (46, 47), allowing random fluctuations and neutral processes to have a greater influence on community dynamics (47–49). However, it should be noted that it is difficult to determine the extinction of microbial taxa in the community, which makes it hard to directly detect drift in the community. The dormancy of microorganisms can prevent them from extinction and weaken the influence of drift, which can also increase the difficulty of directly detecting drift (50). Both the iCAMP approach and the neutral model detected a significant proportion of deterministic assembly factors (e.g., homogeneous selection) (Fig. 4; Table 2), which implies that deterministic processes also play an important role. Thus, even though stochastic processes dominate the potential diazotrophic β-diversity in alpine soils, deterministic processes should not be neglected.

### Aridity alters co-occurrence patterns

Aridity affects co-occurrence patterns (7). For example, the potential diazotrophic co-occurrence networks from semi-arid or arid habitats included more negative correlations than those from humid habitats (Fig. 5), which could indicate greater interspecific competition in high-aridity habitats. Such interspecific competition could be related to the limited C resources of arid habitats, which are characterized by sparse vegetation and low primary productivity due to water deficiency (51). This finding is supported by a previous report of greater competition in bulk soil, when compared to rhizospheric soil, due to reduced C substrate availability (22). Since N fixation is an energy-expensive process, the lower C availability of arid soils may have a more pronounced effect on potential diazotrophic competition than did the humid soil, with the strength of competition increasing with aridity (7, 52). The distribution of microbial taxa depends on both their survival ability and their persistence ability after establishment in a new environment. Interspecific interactions also affect which species occur and how co-occurring species are organized as a community (53). However, our results indicate significant correlations between co-occurrence network topology (i.e., NV, NE, APL, and MOD) and potential diazotrophic β-diversity (Fig. S7). More specifically, greater β-diversity in higher-aridity habitats was associated with greater vertex count and path lengths and reduced connectivity. These findings indicate that co-occurrence patterns are strongly correlated with potential diazotrophic biogeography. Some studies suggest that co-occurrence patterns are a deterministic process that regulates the intensity of intra-species and inter-species competition, generates niche partitioning, and limits community similarity, resulting in high β-diversity (9, 54). However, our results suggest that the contribution of co-occurrence patterns to β-diversity in arid habitats is greater than that of deterministic processes, even community assembly (Fig. 7), indicating that it is inappropriate to include the role of co-occurrence patterns in deterministic processes. Additionally, there are studies indicating that co-occurrence patterns can impact later species colonization through environmental changes or preferential effects (23, 55, 56). Other studies indicate that co-occurrence species participate in various interactions, such as quorum sensing, interference (toxin secretion), and developmental competition (57, 58). The adaptability and population dynamics of individual species are influenced by the abundance changes in the species that directly interact with them (57). This impact, amplified through interaction networks, may serve as a driving factor in shaping community diversity (58). However, our understanding of how co-occurrence patterns, such as commensalism, mutualism, and parasitism, shape microbial communities remains limited (59). This is primarily due to the inherent challenges associated with observing and documenting such patterns in microbial communities (23).

### The relative importance of the stochastic processes and co-occurrence patterns changing with aridity shaped β-diversity

According to the contemporary coexistence theory derived from macroecology, the community β-diversity pattern is shaped by a combination of factors, including the species pool, community assembly, and co-occurrence patterns (9, 10, 34). However, in the present study, the species pool contributed little to the biogeographic patterns of the alpine soil potential diazotrophic communities (Fig. 3), which is inconsistent with our hypothesis (ii). These findings correspond with the results of Qian et al. (60) and Xu et al. (61), who reported that community assembly processes drive β-diversity at a local scale, rather than the species pool, but contrast with the results of Wang et al. (45), who reported that the species pool explains more variations in potential diazotrophic β-diversity over vast biogeographic regions. Thus, the relative contribution of species pools to β-diversity may depend on the organism type or may be influenced by the interplay of multiple ecological mechanisms, such as environmental selection, dispersal limitation, and biological co-occurrence. At large scales, community assembly processes have a greater impact on β-diversity due to changes in spatial and environmental factors, and the importance of co-occurrence patterns increases with decreasing scale (45). The increased effects of assembly processes and co-occurrence patterns could weaken the role of species pools.

Even though both stochastic processes and co-occurrence patterns played significant roles in shaping potential diazotrophic communities in the present study, the relative importance of the stochastic processes and co-occurrence patterns changed with increasing aridity, with the contribution of assembly processes decreasing with increasing aridity and the contribution of co-occurrence patterns increasing (Fig. 6 and 7). This identified our hypothesis (iii). The resource utilization assumption supports that resource competition largely drives community diversification, especially when microbial taxa have similar resource requirements or niches in a resource-poor environment (8). The poor supply of nutrients (e.g., C and N) in arid environments enhances interspecific resource competition, which is, in turn, commonly expected to promote diversity, as evidenced by the more negative correlation in arid microbial networks (52). Co-occurring and competing taxa may stimulate community performance to improve resource use efficiency (62). Soils harboring interspecific competition may contribute to the biogeographic pattern in natural ecosystems (63, 64). Network topology indicated that *Paenibacillus* as keystone taxa were observed exclusively in arid diazotrophic networks (Fig. S6). When the networks were divided into modules, the diazotrophic keystone taxa showed fierce competition with the contacted members in the corresponding modules (20). The presumed keystone taxon (*Paenibacillus*) exhibits competitive traits and advantages for utilizing limited resources more efficiently. It is noteworthy that cooperation or competition does not always correspond to “well” or “poor” ecosystem functioning because higher functionality was observed in a dry ecosystem where fierce microbial competition exists (65–67).

### Conclusion

Our study identified aridity-driven mechanisms that underlie spatial patterns of potential diazotrophic β-diversity in alpine ecosystems. Increasing aridity is associated with reduced β-diversity and low species turnover. Aridity affects the co-occurrence patterns and community assembly of potential diazotrophs by regulating soil and vegetation characteristics. The observed aridity-induced β-diversity patterns were largely affected by the community assembly processes (e.g., dispersal limitation and ecological drift) and co-occurrence patterns, rather than by the species pool, and the relative importance of the stochastic processes (i.e., assembly processes) and co-occurrence patterns decreased and increased, respectively, with increasing aridity. These results provide novel insights into the biogeographical patterns of functional microbes and provide a basis for predicting the response of alpine soil biodiversity and functions to climatic change. However, it is necessary to determine whether this formation mechanism of β-diversity exists at a larger research scale and in other ecosystems and to further explore the effect of this mechanism on soil functions.

## MATERIALS AND METHODS

### Study sites

Field surveys were conducted at 60 sites spanning an aridity gradient (−0.2 to 1.0 aridity level) across the Tibetan Plateau (Fig. S1). Site aridity was represented by 1 − *AI*, where *AI* represents the ratio of precipitation to potential evapotranspiration and was obtained from the Global Aridity Index and Potential Evapotranspiration Climate database version 2 (http://worldclim.org/). The elevation, mean annual precipitation, and mean annual temperature of the study sites ranged from 3,500 to 4,900 m, from 89 to 540 mm, and from 0.3°C to 7.6°C, respectively. The sites were arranged with intervals of 50 to 100 km, and to minimize the effects of human disturbance, the study sites were each located ≥50 km from human habitation and ≥1 km from major roads. In our study, the sites were grouped into humid (aridity < 0.35), semi-arid (0.35 < aridity < 0.8), and arid (aridity > 0.8) habitats based on the aridity level (68), and the corresponding vegetation included alpine meadows (e.g., *Kobresia*, *Stipa*, *Cleistogenes*, and *Astragalus* species), alpine steppe (e.g., *Stipa*, *Kobresia*, *Cleistogenes*, and *Oxytropis* species), and alpine desert steppe (e.g., *Stipa*, *Ajania*, *Draba*, and *Oxytropis* species).

### Soil and vegetation sampling

Soil and vegetation sampling were conducted in August 2020. Six 1 m × 1 m plots were established at each of the 60 sites (23 humid, 21 semi-arid, and 16 arid). In each plot, after recording the vegetation coverage (CO), the height, and the number of plant species richness (PR), the aboveground parts were clipped and dried to obtain the aboveground biomass (AGB), and the roots were washed with tap water and dried to obtain the belowground biomass (BGB). For soil sampling, soil cores were collected from five points (four corners and center) in each plot and then fully blended to produce a composite soil sample, and a total of 360 soils were collected. After roots and stones were removed, each of the composite soil samples was separated into two halves after passing through a 2-mm filter. One part of each soil sample was stored at –80°C for subsequent DNA extraction, and the second part of each soil sample was air-dried to facilitate the measurement of physiochemical properties.

### Soil analysis

Soil moisture (SM) was estimated by oven-drying the samples at 105°C for 24 h. Soil bulk density was then estimated using the soil cores (volume, 100 cm^3^), and clay (<0.002 mm) content was analyzed using a laser particle size analyzer (Mastersizer 2000; Malvern Instruments, Malvern, UK). Soil pH was determined using a pH meter and a soil-to-water ratio of 5:2 (vol/wt). Total nitrogen was measured using the Kjeldahl digestion method, and soil NH_4_^+^-N and NO_3_^−^-N contents were analyzed by a segmented flow autoanalyzer system (AutAnalyel; Bran+Luebbe GmbH, Norderstedt, Germany) after being extracted with 2 mol/L KCl (1:10 wt/vol) (25). Dissolved organic nitrogen, dissolved organic carbon, phosphorus, and soil organic carbon were determined following the method of Wang et al. (69). Soil available phosphorus was measured by molybdate ascorbic acid and a UV spectrophotometer (Camspec, Cambridge, UK). Available potassium was determined using a flame photometer after extraction with 1.0 M ammonium acetate (CH_3_COONH_4_) (25).

### Microbial DNA extraction and real-time qPCR

Microbial DNA was extracted from each of the composite soil samples (0.5 g) using a FastDNA SPIN Kit, according to the instructions of the manufacturer (MP Biomedicals, Cleveland, USA), and the quality and concentration of each of the resulting DNA samples were measured using a NanoDrop 2000 spectrometer (Thermo Scientific, Wilmington, DE, USA). The *nif*H gene was analyzed by high-throughput qPCR on an ABI Prism 7500 Real-Time qPCR system (Applied Biosystems, Foster City, CA, USA) using the primer pairs *nif*H-F (5′-AAAGGYGGWATCGGYAARTCCACCAC-3′) and *nif*H-R (5′-TTGTTSGCSGCRTACATSGCCATCAT-3′) according to the procedures described by Zhang et al. (25). The details of amplification are shown in Appendix S1 in the supplemental material.

### Amplicon sequencing and phylogenetic classification

After DNA extraction, the primers *nif*H-F/*nif*H-R with a 12-bp barcode to identify the different samples were used to amplify the *nif*H gene sequences. The PCR program was as follows: 95°C for 3 min, 35 cycles of 95°C for 30 s, and annealing at 55°C for 30 s, followed by extension at 72°C for 10 min. The PCR products were purified using 2.0% agarose gels and purified using the AxyPrep DNA Gel Extraction Kit (Axygen Biosciences, USA). Purified amplicons were combined at equimolar concentrations and paired-end sequenced (2 × 300 bp) using an Illumina MiSeq platform (Illumina, San Diego, CA, USA) following standard protocols. The resulting sequences were processed using USEARCH v11 to merge paired-end sequences, eliminate primer sequences, and filter out low-quantity sequences (quality score < 20, containing ambiguous nucleotides or not matching the prime). The filtered sequences then were clustered into OTUs with 97% sequence similarity using UPARSE, and taxonomic identities were assigned to the OTUs using the RDP classifier (version 2.2; https://sourceforge.net/projects/rdp-classifier/files/rdp-classifier/rdp_classifier_2.2.zip/download). Before statistical analysis, OTU tables were rarefied to an even number of sequences per sample. The details of sequence quality control, PCR amplification, and taxonomy assignment are described in Appendix S1.

### Ecological processes

A recently proposed assembly approach, entitled infer community assembly mechanisms by phylogenetic bin-based null model analysis (iCAMP), was used to evaluate the contribution of ecological processes of the alpine soil potential diazotrophic community assembly (70). OTUs were first divided into bins based on their phylogenetic relationships created by using FastTree (71). The relative contributions of heterogeneous selection, homogeneous selection, homogenizing dispersal, drift, and dispersal limitation in each bin were measured using the within-bin beta net relatedness index (βNRI) and modified Raup-Crick metric (RC_Bray_). Within each bin, significant deviations (βNRI > +1.96 or βNRI < –1.96) were interpreted as the dominance of heterogeneous and homogeneous selection, respectively. The remaining pairwise comparisons with |βNRI| ≤ +1.96 were divided by RC_Bray_. As opposed to those with RC_Bray_ > +0.95, which were considered dispersal limitation, pairwise comparisons with RC_Bray_ < –0.95 were viewed as homogenizing dispersal. The remaining uncategorized pairwise comparisons were used to estimate the relative importance of ecological drift. To explain the effect of the assembly process on β-diversity, the migration rate and environmental adaptability of the potential diazotrophic community were estimated using a neutral community model and Levin’s niche breadth index (14). The first axes of the PCA based on βNRI (βNRI1) and RC_Bray_ (RC1) were used to represent the community assembly.

### Co-occurrence network construction

Co-occurrence networks of potential diazotrophs in humid, semi-arid, and arid habitats were constructed using the SparCC method (72). Rare OTUs (<20 sequencing reads) were eliminated. Robust and significant Spearman’s correlations (*ρ* > 0.3, *P* < 0.05) were chosen to construct co-occurrence networks. A set of metrics (NE, NV, and positive/negative edge ratio) was calculated to describe the network topologies. The vertices in the networks represent OTUs, and the edges mean Spearman’s associations between vertices (i.e., OTUs). Co-occurrences across samples were represented by the interacted vertices. Subsequently, subnetworks were extracted, and NE, NV, AD, CC, MOD, DEN, and APL were calculated to describe the topology of each subnetwork. The vertices with high *Z*- or *P*-scores were identified as keystone taxa, such as peripherals (Zi < 2.5, Pi < 0.62), connectors (Zi < 2.5, Pi > 0.62), network hubs (Zi > 2.5, Pi > 0.62), and module hubs (Zi > 2.5, Pi < 0.62) (73). An interactive Gephi platform (http://gephi.github.io/) was used to visualize the networks. The first PCA axis of the standardized module expression data was viewed as the network’s module eigengene (74), and the relationships between the module eigengene, soil properties, plant characteristics, assembly processes, and β-diversity were evaluated based on their Spearman’s coefficients.

### Statistical analysis

The Chao1 index and total OTU richness were used to represent potential diazotrophic α-diversity and γ-diversity (species pool), respectively. The first axis of the PCA, which was based on the Bray-Curtis dissimilarity of potential diazotrophic OTUs, was defined as the potential diazotrophic β-diversity. Distance-decay relationships, which represent variation in community structure along a gradient in space or environment, were used to quantify changes in potential diazotrophic β-diversity over geographic distance (45). Distance-decay relationship slopes, which represent spatial species turnover rates, with steeper slopes indicating higher turnover rates, were estimated by the OLSLRM using the vegan package (75), and the significance of distance-decay relationships between habitats was evaluated using the *F* test. Nonmetric multidimensional scaling analysis was performed to evaluate the differences between the compositions of potential diazotrophic communities among the aridity habitats, followed by permutational multivariate analysis of variance tests (*P* < 0.05) using the vegan package (75). A *post hoc* Tukey’s test was used to evaluate the significance of changes in the relative abundance of taxonomic groups along the aridity gradient using the stats package (76). The relationships between α-diversity, β-diversity, and γ-diversity were estimated with the OLSLRM using the stats package (76).

To assess the effect of the potential diazotroph species pool on β-diversity, the expected β-diversity among communities was calculated using an assembly simulation of the communities, as described by Kraft et al. (77). The association between co-occurrence network topologies and β-diversity was evaluated using the Spearman coefficient. Random forest analysis was performed to quantify the relative contributions of various predictors [e.g., soil properties (e.g., pH, soil bulk density, and soil moisture) and plant characteristics (aboveground biomass, belowground biomass, coverage, plant richness, and height)], community assembly (βNRI1 and RC1), and co-occurrence networks (NE, NV, AD, CC, DEN, MOD, and APL) to community β-diversity. Path analysis diagrams using the PLS-PM were used to identify direct and indirect effects of aridity, soil properties, and plant characteristics on potential diazotrophic β-diversity. Each latent variable was chosen according to the values of Cronbach’s alpha, Dillon-Goldstein’s rho, loadings, and cross-loadings, including aridity for climate factors; pH, soil organic carbon, total nitrogen, and SM for soil properties; aboveground biomass, belowground biomass, coverage, and plant richness for vegetation variables; and NV, APL, MOD, and DEN for co-occurrence patterns. Path coefficients represent the direction and strength of the linear relationships between the latent variables and the explained variability (*R*^2^). The goodness of fit was used to assess the predictive power of the established PLS-PM, with a goodness of fit of >0.6 considered acceptable. The PLS-PM was constructed using the “plspm” package (http://www.gastonsanchez.com/PLS_Path_Modeling_with_R.pdf) in R. All analyses were performed using R 4.2.0 (76).

## Data Availability

Raw sequence data reported in this paper have been deposited in the Genome Sequence Archive in the Big Data Center, Chinese Academy of Sciences, under accession number CRA010092 for the *nifH* gene, publicly accessible at bigd. R and Linux scripts used in this study are available from GitHub.

## SUPPLEMENTAL MATERIAL

Abstract msystems.01042-23-s0001.tif Graphical abstract.

Supplemental material msystems.01042-23-s0002.docx Supplemental methods; Fig. S1 to S7.

ASM does not own the copyrights to Supplemental Material that may be linked to, or accessed through, an article. The authors have granted ASM a non-exclusive, world-wide license to publish the Supplemental Material files. Please contact the corresponding author directly for reuse.
