# Supplementary figures and images for: Effect of aridity on the β-diversity of alpine soil potential diazotrophs: insights into community assembly and co-occurrence patterns
Source: mSystems. 2023 Dec 7;9(1):e01042-23. doi: 10.1128/msystems.01042-23 (PMC10804954; doi:10.1128/msystems.01042-23)

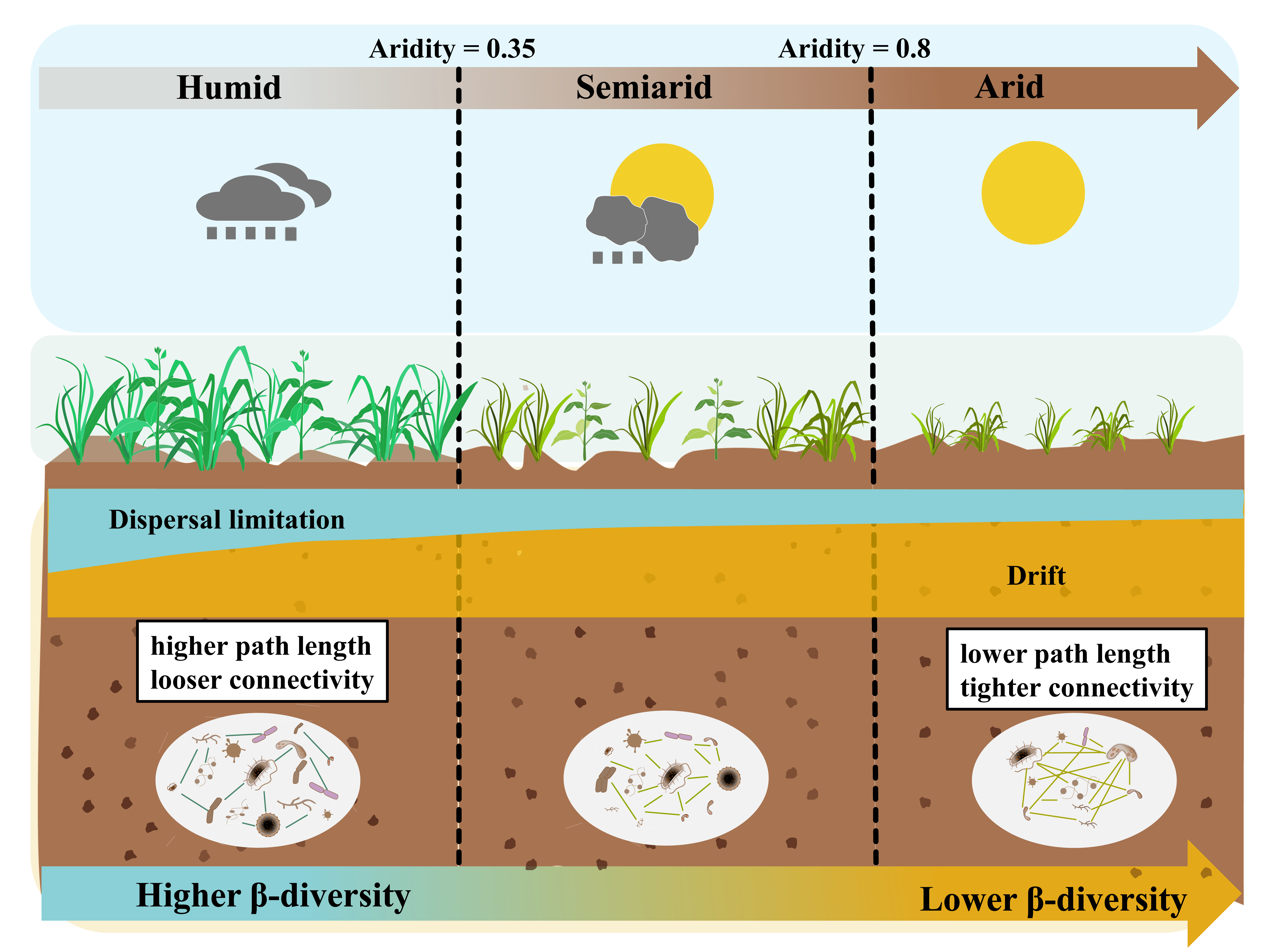

Supplement: Abstract — Graphical abstract. [file msystems.01042-23-s0001.tif]
