# Supplementary material for: Effect of aridity on the β-diversity of alpine soil potential diazotrophs: insights into community assembly and co-occurrence patterns
Source: mSystems. 2023 Dec 7;9(1):e01042-23. doi: 10.1128/msystems.01042-23 (PMC10804954; doi:10.1128/msystems.01042-23)
Supplement: Supplemental material — Supplemental methods; Fig. S1 to S7. [file msystems.01042-23-s0002.docx]

**Supplementary Information**

**Effect of aridity on β-diversity of alpine soil potential diazotrophs: insights into community assembly and co-occurrence patterns**

Shilong Lei^a,b,c^, Xiangtao Wang^f^, Jie Wang^g^, Lu Zhang^d,e^, Lirong Liao^a,b,c^, Guobin Liu^a,b,c^, Guoliang Wang^d,e^, Zilin Song^h^, Chao Zhang^a,b*^

*^a^ The Research Center of Soil and Water Conservation and Ecological Environment, Chinese Academy of Sciences and Ministry of Education, Yangling, Shaanxi 712100, China*

*^b^ Institute of Soil and Water Conservation, Chinese Academy of Sciences and Ministry of Water Resources, Yangling, Shaanxi 712100, China*

*^c^ University of Chinese Academy of Sciences, Beijing 100049, China*

*^d^ State Key Laboratory of Soil Erosion and Dryland Farming on the Loess Plateau, Northwest A&F University, Shaanxi, 712100, P. R. China*

*^e^ Institute of Soil and Water Conservation, Chinese Academy of Science, Shaanxi, 712100, P. R. China*

*^f^ College of Animal Science, Tibet Agriculture and Animal Husbandry University, Nyingchi, 860000, P. R. China*

*^g^ College of Forestry, Guizhou University, Guiyang, 550025, P. R. China*

*^h^ College of Natural Resources and Environment, Northwest A&F University, Shaanxi, 712100, P. R. China*

* Corresponding author: Chao Zhang, *The Research Center of Soil and Water Conservation and Ecological Environment, Chinese Academy of Sciences and Ministry of Education, Yangling, Shaanxi 712100, China*.

Tel/Fax: +86 02987012872, E-mail addresses: [zhangchao1985@nwafu.edu.cn](mailto:zhangchao1985@nwafu.edu.cn)

**This PDF file includes:**

**Appendix S1**

**Supplementary Fig. 1-7**

**Appendix S1**

***Descriptions of quantitative PCR analysis and amplification procedures***：

Microbial DNA was extracted from each of the composite soil samples (0.5 g) using a FastDNA SPIN Kit, according to the instructions of manufacturer (MP Biomedicals, Cleveland, USA), and the quality and concentration of each of the resulting DNA samples were measured using a NanoDrop 2000 spectrometer (Thermo Scientific, Wilmington, DE, USA). All purified DNA samples were stored at ‒80 °C until analysis.

The *nif*H gene was analyzed by high-throughput qPCR on an ABI Prism 7500 Real-Time quantitative PCR (*q*PCR) system (Applied Biosystems, Foster City, CA, USA) using primer pairs *nif*H-F (5′-AAAGGYGGWATCGGYAARTCCACCAC-3′) and *nif*H-R (5′-TTGTTSGCSGCRTACATSGCCATCAT-3′). The qPCR reaction mixture (20 μL) consisted of 10 μL SYBR^®^ Premix Ex Taq^™^ (Takara Bio USA), 1 μL DNA template, 0.8 μL each of the forward and reverse primers, and 7.4 μL double-distilled water (ddH2O). The qPCR program ran was: denaturation at 98°C for 5 min, then 35 cycles of denaturation at 95°C for 30 s, annealing at 58°C for 30 s, and extension at 72°C for 60 s. Each sample's reactions were done in triplicate, and concurrently negative controls (without DNA templates) were used. Standard curves were produced using *nif*H plasmids at tenfold serial dilutions. The *nif*H gene was quantified based on average slopes obtained from standard curves.

***Amplicon sequencing and phylogenetic classification***

Community composition and diversity of potential diazotrophs were determined by Illumina MiSeq sequencing technology (Illumina Inc.) with the primer sets *nif*H-F/*nif*H-R. The 20 μL PCR reaction mixture consisted of 4 μL 5 × FastPfu buffer, 2 μL dNTPs (2.5 mM), 0.8 μL of each primer (5 μM), 0.4 μL FastPfu polymerase, 0.2 μL bovine serum albumin, 10 ng template DNA, and ddH_2_O. The PCR reactions were performed using TransGen AP221-02 TransStart^®^ FastPfu DNA Polymerase (Transgen Biotech Co., Ltd.) on an ABI GeneAmp^®^ 9700 PCR System (Applied Biosystems). The PCR program was: initial denaturation at 95°C for 3 min, followed by 35 cycles at 95°C for 30 s, annealing at 55°C for 30 s, 72°C for 45 s, and a final extension at 72°C for 10 min. Each sample was amplified three times technically, and the PCR result was then pooled. PCR amplicons were recovered by agarose gel electrophoresis after being purified (1.5% gel agar, 90 V, 45 min). The product was quantified using a QuantiFluor^™^-ST Solid Standard (Promega, Madison, WI, United States), then equimolar concentrations of PCR product for each sample were pooled. Samples were spiked with a 20% (v/v) PhiX control library after being diluted to the appropriate loading concentration for a MiSeq run, and sequenced using the Illumina MiSeq PE300 platform (Majorbio Company in Shanghai, China), producing 2 × 300bp long reads. The instrument was used to demultiplex the reads.

**
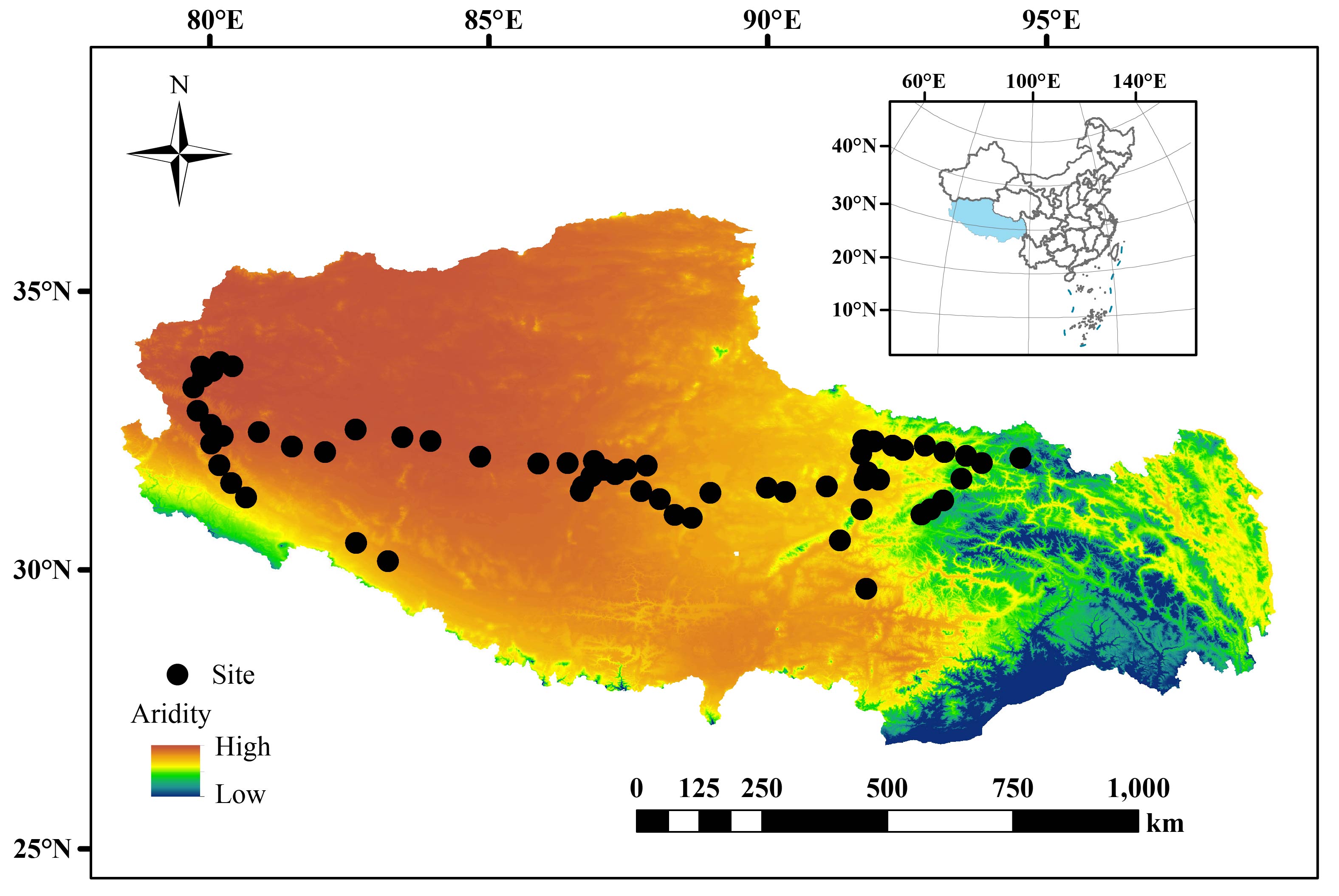
Fig. S1** Sampling sites in this study.

**
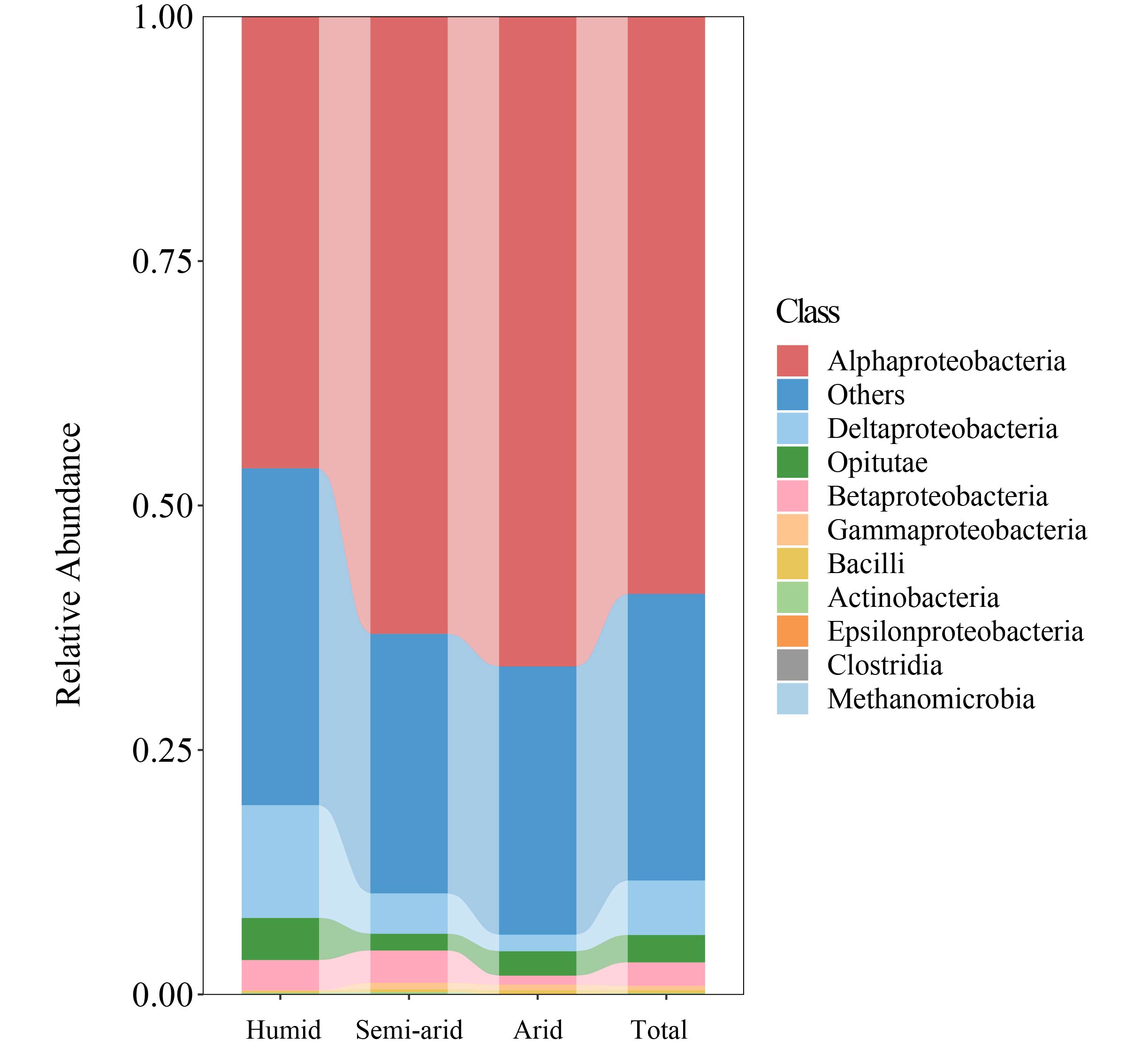
Fig. S2** The relative abundance of potential diazotrophic class in three aridity habitats.

**Aridity = 0.2**


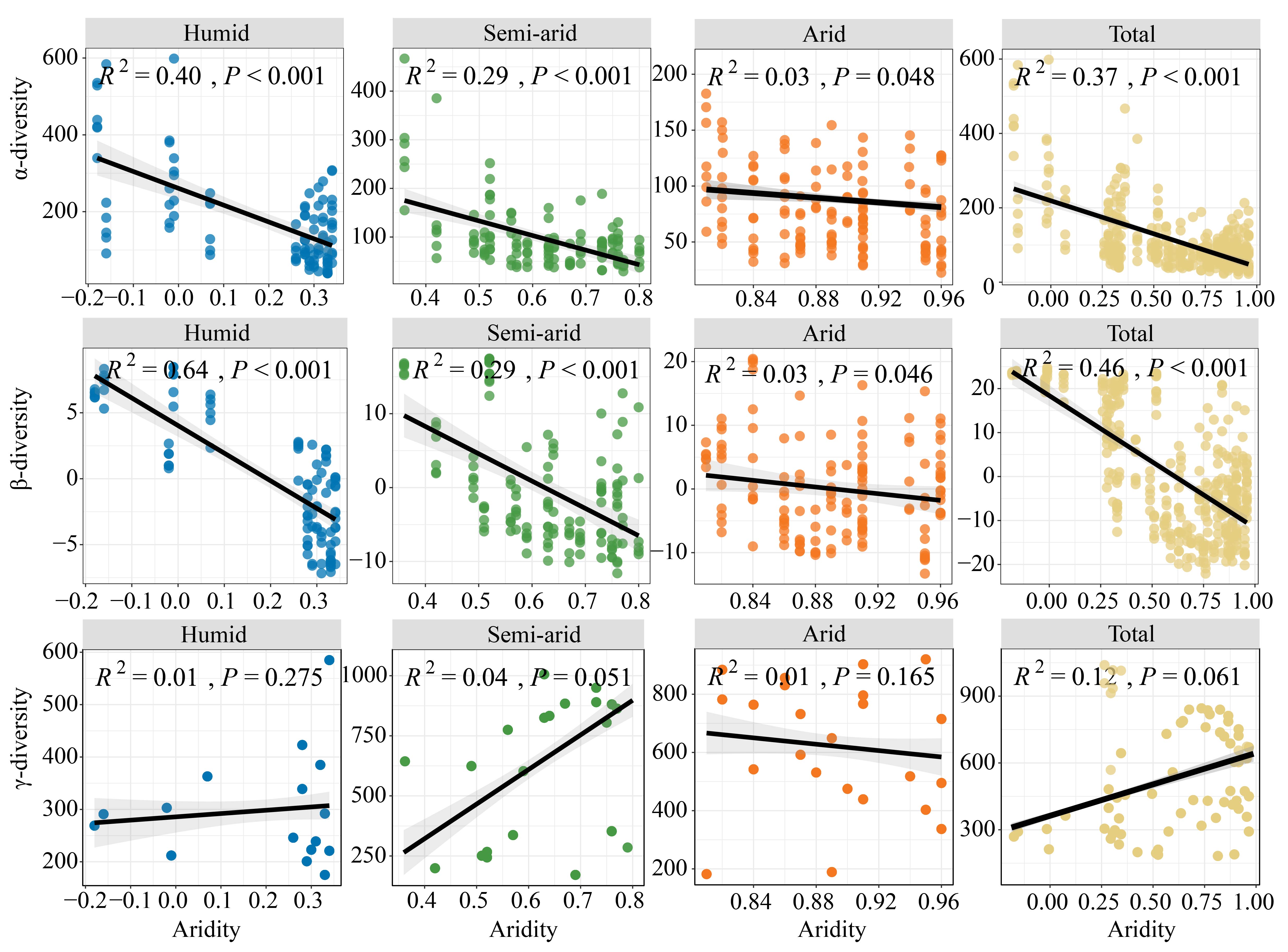
**Fig. S3** Relationships between potential diazotrophic α-, β-, γ-diversity and aridity in different aridity habitats. Grey shading areas around lines indicate 95% confidence intervals of fit.

**
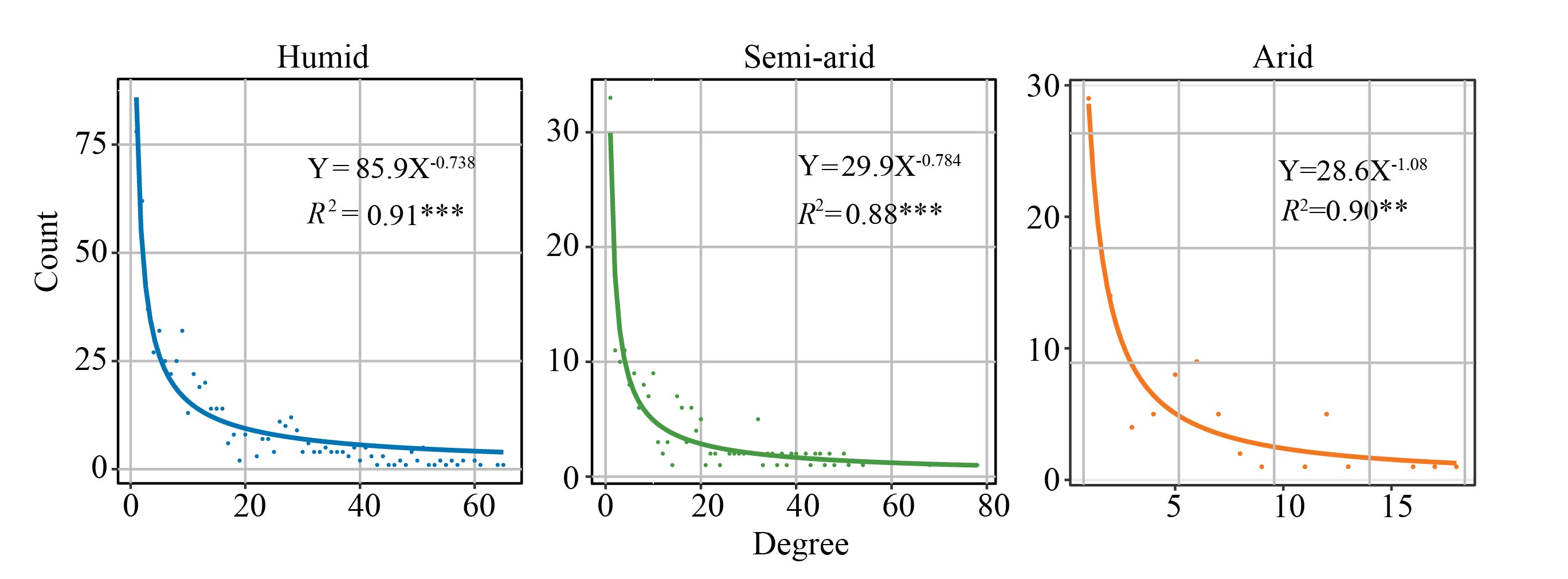
Fig. S4** Relationships between the degree and count of vertices of potential diazotrophic co-occurrence networks in humid-, semiarid- and arid habitats. ^**^: *P* < 0.01; ^***^: *P* < 0.001.

**
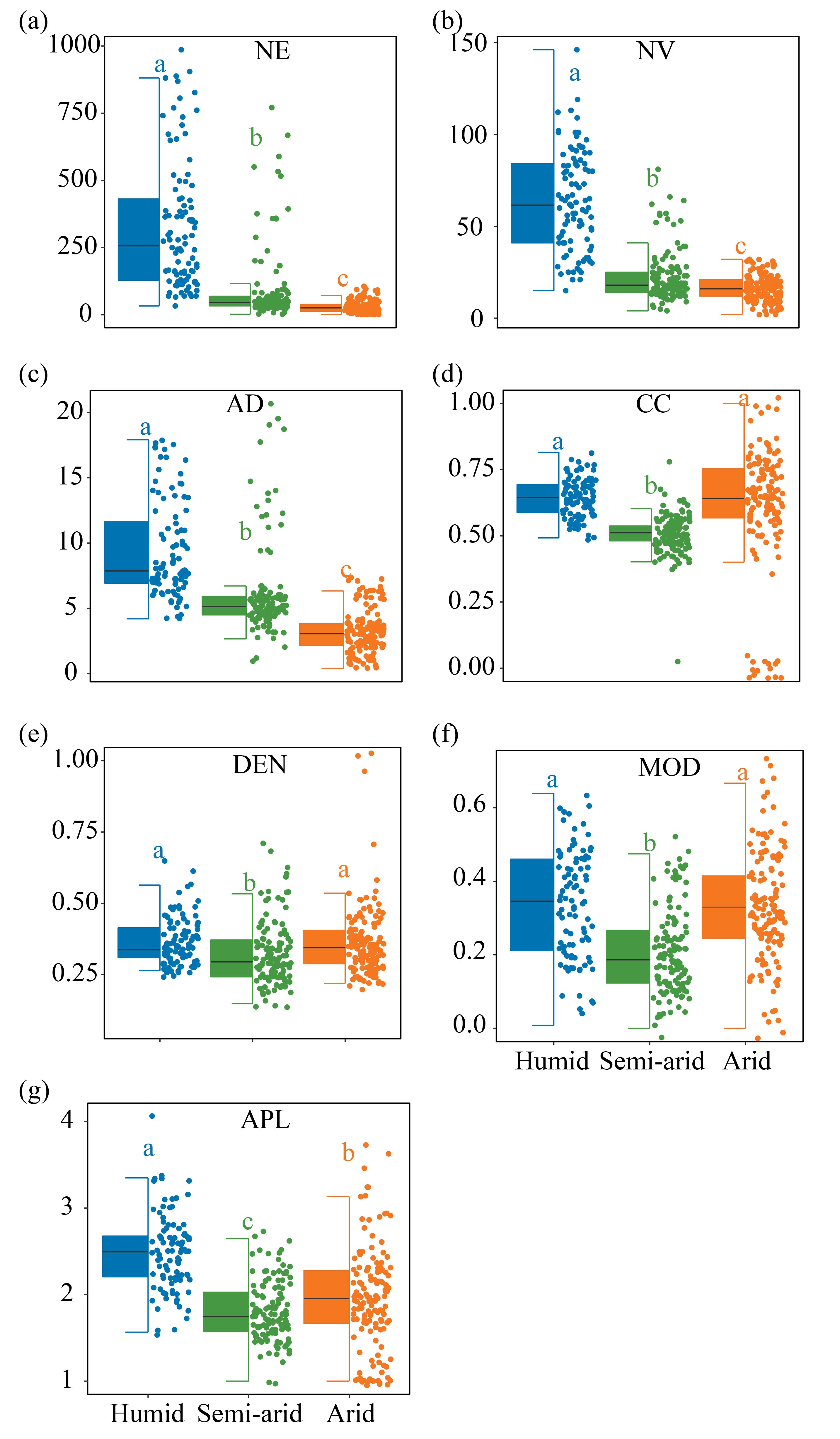
Fig. S5** Topological feature of potential diazotrophic subnetworks. In the box plots, the upper boundary of each box indicates the 25th percentile, the horizontal line inside each box marks the median, and the lower boundary of the box indicates the 75th percentile. Letters indicate significant differences (*P* < 0.05). Different letters indicate significant differences (*P* < 0.05) by Tukey’s test. NE: the number of edges; NV: the number of vertices; AD: average degree; CC: clustering coefficient; MOD: modularity; APL: average path length; DEN: density.

**
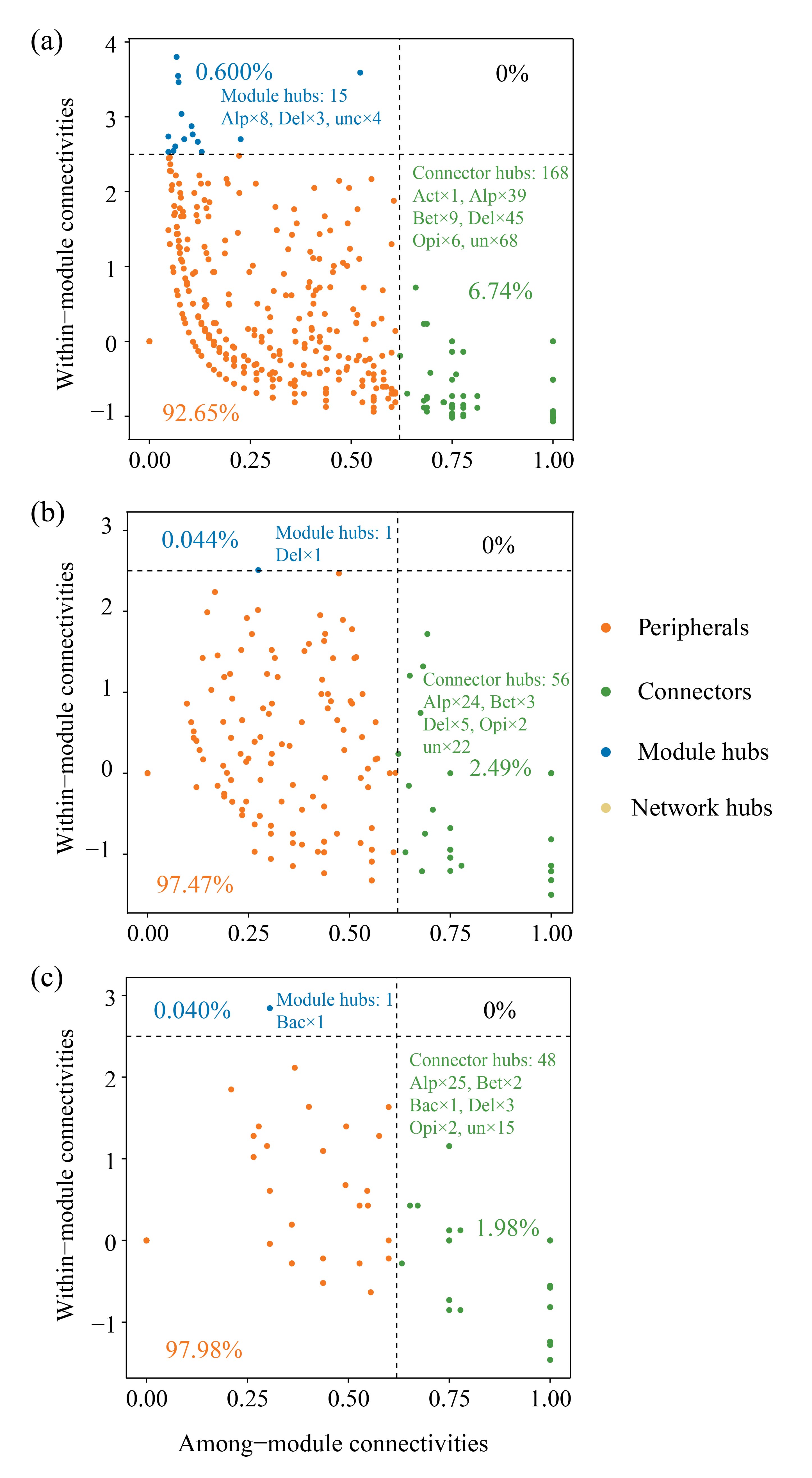
Fig. S6** The topological roles of vertices in humid- (a), semi-arid- (b) and arid (c) habitats. Alp: Alphaproteobacteria; Del: Deltaproteobacteria; unc: unclassified; Act: Actinobacteria; Bet: Betaproteobacteria; Opi: Opitutae; Bac: Bacilli.

**
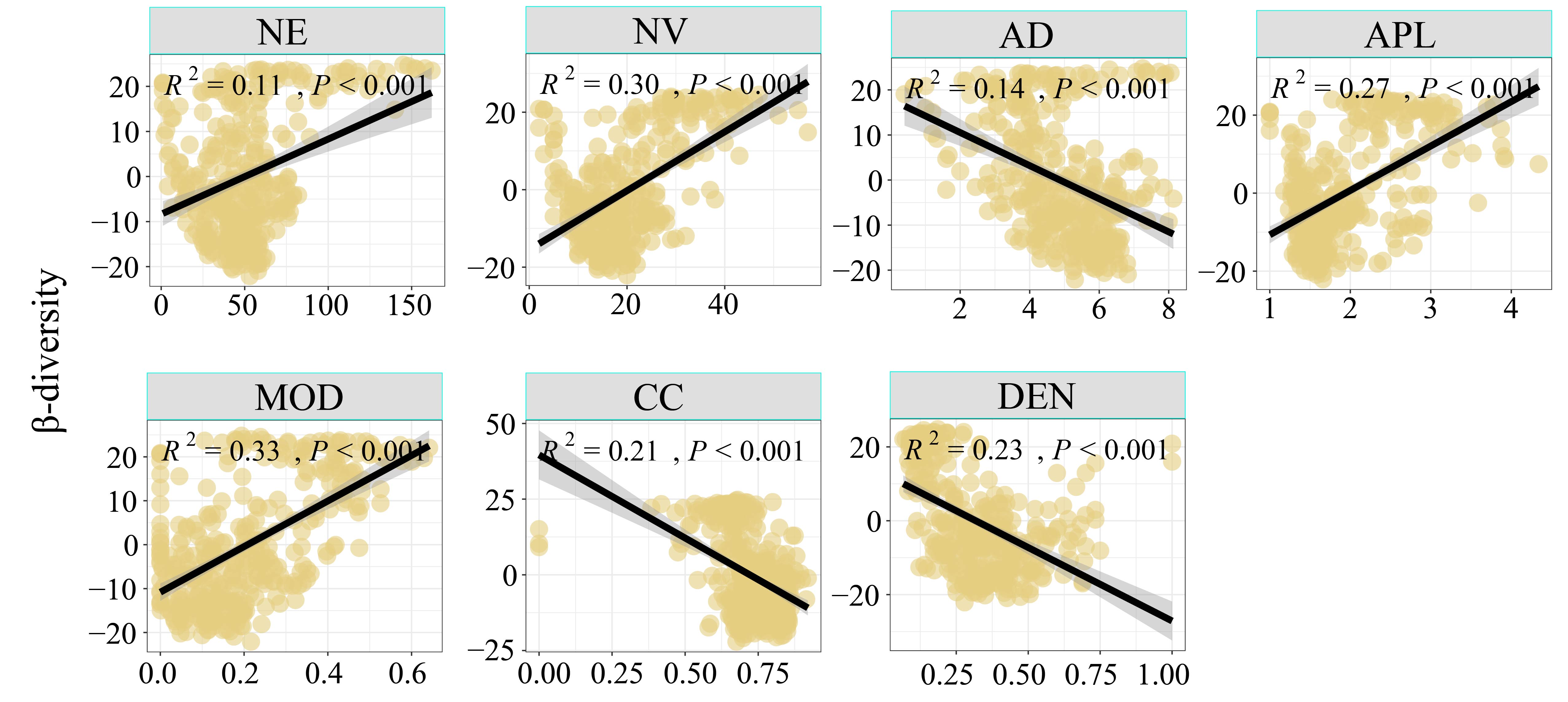
Fig. S7** Relationships between β-diversity of potential diazotrophic community and co-occurrence network topological features. Grey shading areas around lines indicate 95% confidence intervals of fit. NE: the number of edges; NV: the number of vertices; AD: average degree; APL: average path length; MOD: modularity; CC: clustering coefficient; DEN: density.
